# Association between serum uric acid-to-creatinine ratio and non-alcoholic fatty liver disease: a cross-sectional study in Chinese non-obese people with a normal range of low-density lipoprotein cholesterol

**DOI:** 10.1186/s12876-022-02500-w

**Published:** 2022-09-14

**Authors:** Xiaoyu Wang, Yong Han, Yufei Liu, Haofei Hu

**Affiliations:** 1 Department of Nephrology, Hechi People’s Hospital, Hechi, 547000 Guangxi Zhuang Autonomous Region China; 2 Department of Emergency, Shenzhen Second People’s Hospital, Shenzhen, 518000 Guangdong Province China; 3 Department of Emergency, The First Affiliated Hospital of Shenzhen University, Shenzhen, 518000 Guangdong Province China; 4 Department of Neurosurgery, Shenzhen Second People’s Hospital, Shenzhen, 518000 Guangdong Province China; 5 Department of Neurosurgery, The First Affiliated Hospital of Shenzhen University, Shenzhen, 518000 Guangdong Province China; 6 Department of Nephrology, Shenzhen Second People’s Hospital, No. 3002 Sungang Road, Futian District, Shenzhen, 518000 Guangdong Province China; 7 Department of Nephrology, The First Affiliated Hospital of Shenzhen University, Shenzhen, 518000 Guangdong Province China; 8 Shenzhen University Health Science Center, Shenzhen, 518000 Guangdong Province China

**Keywords:** Non-alcoholic fatty liver disease, Serum uric acid-to-creatinine ratio, Cross-sectional study, Non-linear, Generalized additive model, Binary logistic regression model

## Abstract

**Objective:**

Evidence regarding the relationship between serum uric acid-to-creatinine (SUA/Scr) ratio and non-alcoholic fatty liver disease (NAFLD) in Chinese non-obese people is still limited. Therefore, the present study was undertaken to analyze the association between the SUA/Scr ratio and NAFLD.

**Methods:**

This study was a cross-sectional study that non-selectively and consecutively collected 182,320 non-obese individuals with a normal range of low-density lipoprotein cholesterol in a Chinese hospital from January 2010 to December 2014. A binary logistic regression model was used to evaluate the independent association between the SUA/Scr ratio and NAFLD. A generalized additive model (GAM) and smooth curve fitting (penalized spline method) was conducted to explore the exact shape of the curve between them. A series of sensitivity analyses were used to ensure the robustness of the results. Moreover, subgroup analyses were conducted. In addition, the diagnostic value of the SUA/Scr ratio for NAFLD was evaluated based on the area under the receiver-operating characteristic curve (AUROC). It was stated that the data had been uploaded to the DATADRYAD website.

**Results:**

The average participants' age was 40.96 ± 14.05 years old, and 90,305 (49.5%) were male. The prevalence of NAFLD was 13.7%, and the mean SUA/Scr was 3.62 ± 0.92. After adjusting covariates, the results showed that SUA/Scr ratio was positively associated with NAFLD (OR = 1.424, 95%CI: 1.396, 1.454). There was also a non-linear relationship between SUA/Scr ratio and NAFLD in participants with normal kidney function, and the inflection point of the SUA/Scr ratio was 4.425. The effect sizes (OR) on the left and right sides of the inflection point were 1.551 (1.504, 1.599) and 1.234 (1.179, 1.291), respectively. And the sensitive analysis demonstrated the robustness of the results. Subgroup analysis showed a stronger association between SUA/Scr ratio and NAFLD in females and the population with age < 50 years, FPG ≤ 6.1 mmol/L, BMI < 24 kg/m^2^, and HDL-c ≥ 1 mmol/L. In contrast, the weaker association was probed in males and the population with age ≥ 50 years, BMI ≥ 24 kg/m^2^, FPG > 6.1 mmol/L, and HDL-c < 1 mmol/L. The SUA/Scr ratio had an AUC of 0.6624 (95% CI 0.6589, 0.6660) for diagnosing NAFLD. Based on the best cut-off value of 3.776, the negative predictive value of the SUA/Scr ratio for identifying NAFLD was 91.0%.

**Conclusion:**

This study demonstrates an independent positive association between SUA/Scr ratio and NAFLD in Chinese non-obese people with a normal range of low-density lipoprotein cholesterol. There is also a non-linear relationship between the SUA/Scr ratio and NAFLD in participants with normal kidney function, and the SUA/Scr ratio is strongly related to NAFLD when SUA/Scr ratio is less than 4.425. The SUA/Scr ratio has a certain reference value for determining NAFLD. When the SUA/Scr ratio is lower than 3.776, identifying NAFLD patients with low risk is a great reference.

**Supplementary Information:**

The online version contains supplementary material available at 10.1186/s12876-022-02500-w.

## Background

Non-alcoholic fatty liver disease (NAFLD) represents a series of liver damage processes ranging from simple steatosis to non-alcoholic steatohepatitis (NASH), which can progress further to cirrhosis, liver failure, and hepatocellular carcinoma [1]. Therefore, NAFLD is a significant health hazard and economic and social burden [2]. The estimated prevalence rate of NAFLD ranges from 25 to 31% in the general population of China in recent years [3, 4]. NAFLD is an independent risk factor for chronic kidney disease (CKD), diabetes, and cardiovascular disease [5–9]. Furthermore, patients with severe chronic diseases such as type 2 diabetes (T2DM), cardiovascular disease (CVD), chronic liver disease, and CKD are at increased risk for NAFLD [10–13]. In addition, NAFLD is considered a hepatic component of metabolic syndrome as it is highly associated with overweight, dyslipidemia, obesity, hyperglycemia, insulin resistance, hypertension, and T2DM [14].

Clinically, obesity is closely related to NAFLD [10, 15, 16]. However, it is worth noting that many persons with a normal body mass index (BMI) are still diagnosed with NAFLD in the general population. 7.4% of non-obese adults could be diagnosed with hepatic steatosis by ultrasound in the Third National Health and Nutrition Inspection Survey of America [17]. In Asia, this figure can be as high as 8–19% [18]. Besides, more studies have shown that non-obese patients with NAFLD appear more inclined to metabolic syndrome and progress to severe liver disease more rapidly [19, 20]. In addition, early detection of non-obese NAFLD can reduce the risk of diabetes and cardiovascular disease [21–23]. Therefore, identifying non-obese individuals at risk of NAFLD may still be essential. Dyslipidemia is a comorbidity of NAFLD [24]. Moreover, many studies have proved that low-density lipoprotein cholesterol (LDL-c) is associated with NAFLD [25, 26]. Meanwhile, a recent study suggested that elevated LDL-c levels within the normal range might play an important role in the incidence and prevalence of NAFLD [27]. The rising prevalence and complexity of NAFLD in China require our persistent efforts to find new risk factors for prevention and treatment.

Uric acid (UA) is the end product of purine metabolism derived from endogenous and exogenous sources [28]. Serum uric acid (SUA) levels are maintained by the balance between uric acid production and excretion, and hyperuricemia is caused by disruption of this balance [29]. High SUA levels are often associated with hyperlipidemia, metabolic syndrome, hypertension, diabetes, and CKD [30], all contributing to CVD progression. A meta-analysis shows an association between SUA and NAFLD, suggesting that elevated SUA levels may prompt physicians to screen for NAFLD [31].

More recently, studies have shown that higher serum uric acid to creatinine ratio (SUA/Scr ratio) is associated with increased metabolic syndrome risk [32] and predicts incident CKD in T2DM patients [33] and patients with chronic obstructive pulmonary disease(COPD) compared with SUA alone [34]. However, only a few studies focus on the relationship between the SUA/Scr ratio and NAFLD by reviewing the medical literature. As SUA excretion is mainly affected by renal function, it is acceptable that renal function-normalized SUA could reflect the endogenic UA level precisely [35]. Gu et al. demonstrated that the SUA/Scr ratio might be superior to SUA only as a predictor of CKD in T2DM patients [33]. A recent cross-sectional study including 778 subjects who participated in a health checkup showed that NAFLD participants had higher levels of SUA/Scr ratio than those without NAFLD and that the prevalence of NAFLD increased with the level of the SUA/Scr ratio, suggesting a significant association between the SUA/Scr ratio and NAFLD (OR = 1.182, 95% CI: 1.066–1.311) [25]. Another cross-sectional study included 282 individuals with normal SUA levels and different glucose tolerance statuses [36]. Logistic regression analysis suggested that the SUA/Scr ratio was positively associated with NAFLD (OR = 1.548, 95% CI: 1.018–2.352). Unfortunately, neither study performs subgroup analyses nor explores the non-linear relationship between the SUA/Scr ratio and NAFLD. Besides, the diagnostic value of the SUA/Scr ratio for NAFLD is also unclear. In addition, the current study is limited by the small sample size. The link between the SUA/Scr ratio and NALFD has not yet been widely explored among non-obese populations with a normal range of low-density lipoprotein cholesterol in China. Based on the reason mentioned above, a cross-sectional study was designed in a large Chinese non-obese population with a normal range of LDL-c. This study was mainly based on the following purposes: (1) To analyze the independent linear relationship of the SUA/Scr ratio and NAFLD; (2) To explore the possible non-linear relationship between the SUA/Scr ratio and NAFLD; (3) Subgroup analysis of differences in the relationship between the SUA/Scr ratio and NAFLD in different populations (such as impaired kidney function and normal kidney function); (4) To analyze the diagnostic value of the SUA/Scr ratio for NAFLD.

## Methods

### Study design

The present cross-sectional study was conducted using records from a computerized database established by the Wenzhou Medical Center of Wenzhou People’s Hospital in China. The interesting independent variable in the present work was the serum uric acid-to-creatinine ratio. The dependent variable was NAFLD (dichotomous variable: NAFLD, non-NALFD).

### Data source

The raw data was downloaded freely from the DATADRYAD database (https://datadryad.org/stash) provided by Sun et al. [26]. From: Association of low-density lipoprotein cholesterol within the normal range and NAFLD in the non-obese Chinese population: a cross-sectional and longitudinal study. Dryad Digital Repository. http://dx.doi.org/ 10.1136/bmjopen-2016–013,781. Under Dryad's terms of service, researchers could use this data for secondary analyses without violating authors' rights.

### Study population

The original researchers non-selectively and consecutively collected Chinese people who participated in a health examination at Wenzhou Medical Center of Wenzhou People’s Hospital [26]. Participants' identities were encoded into untraceable codes to alleviate potential privacy concerns in this study. The clinical data of participants were acquired and stored in the electronic data capture system. This research was conducted under the approval of the Clinical Research Ethics Committee in Wenzhou People’s Hospital. All participants gave written informed consent to participate [26].

**Fig. 1:**
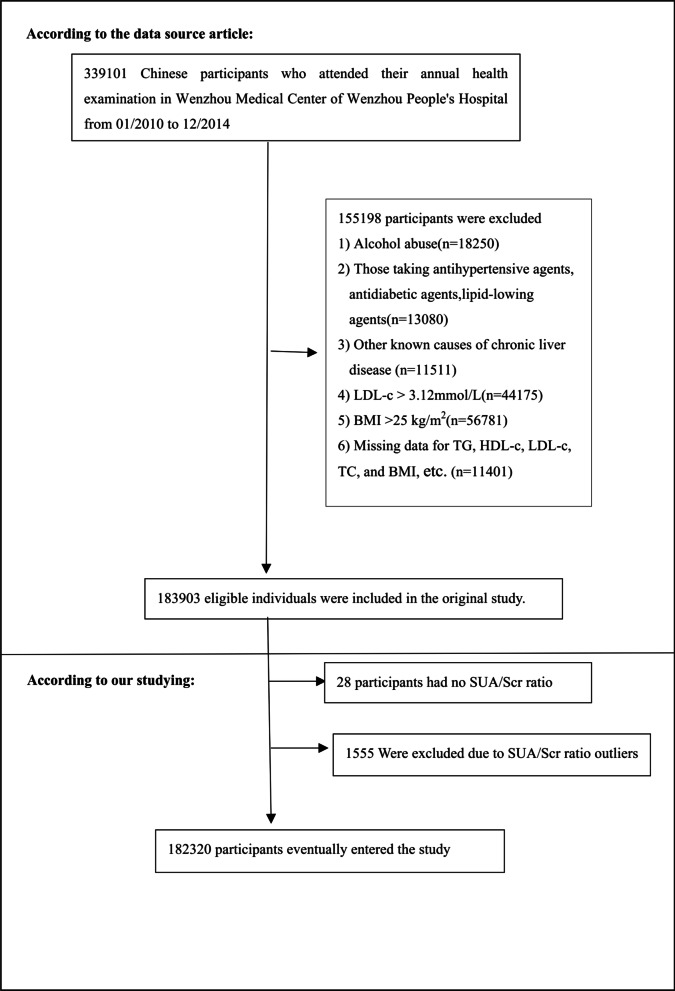
Flowchart of study participants

The original study initially enrolled 339,101 Chinese individuals [26]. Afterward, 156,781 participants were excluded, and left 182,320 participants were left for data analysis (see flowchart for details in Fig. 1). The participants' time started and ended in January 2010 and December 2014, respectively. Individuals were excluded if they met any of the following criteria [26]: (1) those with excessive alcohol consumption (per week ≥ 140 g for males and ≥ 70 g/week for females); (2) Those with any known causes of chronic hepatic diseases, such as autoimmune or viral hepatitis; (3) Those with body mass index (BMI) ≥ 25 kg/m^2^ and low-density lipoprotein cholesterol (LDL-c) > 3.12 mmol/L; (4) Those taking antihypertensive, lipid-lowering, or antidiabetic agents; (5) Participants with missing data on total cholesterol (TC), high-density lipoprotein cholesterol (HDL-c), LDL-c, triglyceride (TG), and BMI, etc.; (5) Those had no SUA and Scr records; and (7) Those with the SUA/Scr ratio outliers (out of the range of means plus or minus three standard deviations) [37, 38].

### Variables

#### Serum uric acid-to-creatinine ratio

The serum uric acid-to-creatinine ratio was treated as a continuous variable. The detailed process of the SUA/Scr ratio was described as follows: SUA/Scr ratio = serum uric acid divided by serum creatinine.

#### NAFLD

The interesting outcome variable was NAFLD (dichotomous variable: 1 = NAFLD, 0 = non-NALFD). The detailed procedure for measuring NAFLD was described below: participants were diagnosed with NAFLD by ultrasonography, as recommended by the Chinese Liver Disease Association [39]. Specifically, there were five diagnostic criteria for NAFLD. (1) Diffusely enhanced near-field echoes in the liver region and gradually attenuated far-field echoes. (2) The intrahepatic cavity structure was unclear. (3) Mild to moderate hepatomegaly with a round blunt edge. (4) Reduced blood flow signal in the liver. (5) Blurred or incomplete visualization of the right hepatic lobe and diaphragm [26].

#### Covariates

Our study selected covariates according to our clinical experience and the previous literature. Based on the above principles, therefore, the following variables were used as covariates [26]: (1) continuous variables: age, BMI, alanine aminotransferase (ALT), globulin (GLB), HDL-c, aspartate aminotransferase (AST), albumin (ALB), fasting plasma glucose (FPG), TC, total bilirubin (TB), TG, blood urea nitrogen (BUN), LDL-c, γ-glutamyl transpeptidase (GGT), and direct bilirubin (DBIL); (2) categorical variables: sex. Using standard methods, all the biochemical values were measured by an automated analyzer (Abbott AxSYM). A physician took a health habit inventory and medical history [26]. BMI was calculated as weight in kilograms divided by height in meters square (kg/m^2^). The evaluated glomerular filtration rate (eGFR) was calculated based on age, gender, and Scr, according to the CKD-EPI equation [40]. Data were collected under standardized conditions and processed according to uniform procedures. According to the World Health Organization (WHO), impaired fasting glucose (IFG) was defined as FPG of 6.1–6.9 mmol/L [41]. FPG ≥ 7 mmol/L was defined as diabetes [42]. ALT > 40 U/L reflected liver dysfunction [43]. Impaired kidney function defined as estimated glomerular filtration rate (eGFR) < 60 ml/min/1.73m^2^ [44]. More specific details were provided in previous reports [26, 45, 46].

#### Statistical analysis

The participants were stratified by quintiles of the SUA/Scr ratio. Mean ± standard deviation (SD) (Gaussian distribution) or median (interquartile ranges) (Skewed distribution), were reported for continuous variables, and frequencies and percentages were presented for categorical variables. We used χ2 (categorical variables), One-Way ANOVA test (normal distribution), or Kruskal-Whallis H test (skewed distribution) to test for differences among different SUA/Scr ratio groups.

The number of participants with missing data of ALT, GGT, AST, ALB, TP, GLB, DBIL, TB, BUN, and FPG were 30,389 (16.7%), 30,389 (16.7%), 30,389 (16.7%), 23,630 (13.0%), 23,629 (13.0%), 23,629 (13.0%), 72,282 (39.6%), 58,751 (32.2%), 2 (0.001%), and 145 (0.08%), respectively. Multiple imputations were used to handle the missing data [47]. The imputation model included age, sex, BMI, AST, ALB, ALT, GLB, HDL-c, DBIL, BUN, TG, GGT, FBG, TC, TB, and LDL-c. Missing data analysis procedures used missing-at-random (MAR) assumptions [48].

To achieve the research purpose of this study, we mainly performed statistical analysis from the following aspects.

##### To analyze the independent linear relationship of the SUA/Scr ratio and NAFLD

After collinearity screening, three different models were built using univariate and multivariate binary logistic regression models according to the STROBE statement [49], including a non-adjusted model (Crude model: no covariates were adjusted), a minimally-adjusted model (Model I: only sociodemographic variables were adjusted, including age, gender, and BMI), and a fully-adjusted model (Model II: covariates presented in Table 1 were adjusted, including age, sex, BMI, AST, ALB, ALT, GLB, GGT, HDL-c, DBIL, BUN, TG, FBG, TB, eGFR and LDL-c). Effect sizes (OR) with 95% confidence intervals were recorded. We adjusted them when the covariances were added to the model, and the odds ratio (OR) changed by 10% or greater [49]. Also, it referred to the results of the collinearity screening. According to the results of the collinearity screening, TC was collinear with other variables, so we did not finally include TC in the multivariate logistic regression equation.

**Table 1:** The baseline characteristics of participants

SUA/Scr Quintile	Q1(< 2.8)	Q2(≥ 2.8 to < 3.3)	Q3(≥ 3.3 to < 3.8)	Q4(≥ 3.8 to < 4.3)	Q5(≥ 4.3)	*P*-value
N	36,464	36,458	36,459	36,471	36,468	
Age (years) Gender Female Male	42.1 ± 14.0 24,013 (65.9%) 12,451 (34.1%)	41.0 ± 14.2 19,924 (54.6%) 16,534 (45.4%)	40.6 ± 14.1 17,540 (48.1%) 18,919 (51.9%)	40.5 ± 14.0 15,860 (43.5%) 20,611 (56.5%)	40.6 ± 13.9 14,678 (40.2%) 21,790 (59.8%)	< 0.001 < 0.001
BMI (kg/m^2^)	20.9 ± 2.1	21.2 ± 2.1	21.5 ± 2.1	21.6 ± 2.1	21.9 ± 2.1	< 0.001
GGT (U/L)	18.0 (14.0–25.0)	19.0 (14.0–27.9)	21.0 (15.0–30.4)	22.0 (16.0–34.0)	26.0 (17.0–42.8)	< 0.001
ALT (U/L)	15.0 (11.0–21.0)	15.0 (11.0–22.0)	16.0 (12.0–23.5)	17.0 (12.0–25.0)	19.0 (12.9–28.0)	< 0.001
AST (U/L)	20.0 (17.0–24.0)	20.0 (17.0–24.0)	21.0 (17.6–25.0)	21.0 (18.0–26.0)	22.0 (18.0–27.0)	< 0.001
ALB (g/L)	44.0 ± 2.7	44.3 ± 2.7	44.6 ± 2.7	44.8 ± 2.6	45.2 ± 2.6	< 0.001
GLB (g/L)	29.4 ± 3.7	29.4 ± 3.7	29.3 ± 3.6	29.3 ± 3.7	29.3 ± 3.6	0.001
TB (umol/L)	11.9 ± 4.9	12.3 ± 4.9	12.4 ± 5.1	12.5 ± 5.1	12.4 ± 5.2	< 0.001
DBIL (umol/L)	1.8 (1.3–2.5)	1.8 (1.3–2.5)	1.8 (1.3–2.5)	1.9 (1.3–2.6)	1.9 (1.3–2.7)	< 0.001
BUN (mmol/L)	4.3 ± 1.5	4.3 ± 1.2	4.4 ± 1.2	4.4 ± 1.2	4.5 ± 1.2	< 0.001
Scr (umol/L)	81.4 ± 26.1	80.5 ± 17.8	79.8 ± 17.1	78.1 ± 16.7	73.3 ± 16.3	< 0.001
eGFR (ml/min/1.73m^2^)	94.2 ± 19.2	97.3 ± 17.7	99.7 ± 17.3	102.8 ± 17.1	108.6 ± 16.9	< 0.001
UA (umol/L)	197.5 ± 55.4	248.4 ± 55.9	281.9 ± 61.1	314.9 ± 68.1	365.7 ± 83.8	< 0.001
SUA/Scr ratio	2.4 ± 0.3	3.1 ± 0.1	3.5 ± 0.1	4.0 ± 0.2	5.0 ± 0.5	< 0.001
FBG (mmol/L)	5.2 ± 0.9	5.2 ± 0.8	5.1 ± 0.8	5.1 ± 0.8	5.1 ± 0.9	< 0.001
TC (mmol/L)	4.5 ± 0.7	4.5 ± 0.7	4.5 ± 0.7	4.6 ± 0.7	4.6 ± 0.8	< 0.001
TG (mmol/L)	0.9 (0.7–1.2)	1.0 (0.8–1.4)	1.1 (0.8–1.5)	1.1 (0.8–1.7)	1.3 (0.9–2.0)	< 0.001
HDL-c (mmol/L)	1.6 ± 0.4	1.5 ± 0.3	1.4 ± 0.3	1.4 ± 0.3	1.3 ± 0.4	< 0.001
LDL-c (mmol/L)	2.2 ± 0.5	2.2 ± 0.5	2.3 ± 0.5	2.3 ± 0.5	2.3 ± 0.5	< 0.001

Values are n (%) or mean ± SD or median (quartile)

*ALB* albumin; *ALT* alanine aminotransferase; *AST* aspartate aminotransferase; *BMI* body mass index; *BUN* blood urea nitrogen; *Scr* serum creatinine; *DBIL* direct bilirubin; *TB* total bilirubin; *FPG* fasting plasma glucose; *GGT* γ-glutamyl transpeptidase; *GLB* globulin; *HDL-c* high-density lipoprotein cholesterol; *LDL-c* low-density lipoprotein cholesterol; *TC* total cholesterol; *TG* triglyceride; *UA* uric acid; *SUA/Scr ratio* serum uric acid-to-creatinine ratio; *eGFR* estimated glomerular filtration rate

##### To analyze the non-linear relationship of the SUA/Scr ratio and NAFLD

Since methods based on binary logistic regression models were often suspected of being incapable of handling nonlinear models, generalized additive models (GAM) and smooth curve fitting (penalized spline method) were used to address the nonlinear relationship between the SUA/Scr ratio and NAFLD. If nonlinearity was detected, we first calculated the inflection point using a recursive algorithm, then built a two-piece binary logistic regression model on either side of the inflection point [50]. A log-likelihood ratio test was used to determine the most appropriate model describing the association between the SUA/Scr ratio and NAFLD.

##### Subgroup analysis

The subgroup analyses were conducted using the stratified binary logistic regression model across various subgroups (including gender, FPG, eGFR, age, BMI, HDL-c, ALT, and GLB). Firstly, continuous variable age (< 30, ≥ 30 to < 40, ≥ 40 to < 50, ≥ 50 to < 60, ≥ 60 to < 70, ≥ 70 years), BMI (< 18.5, ≥ 18.5 to < 24, ≥ 24 kg/m^2^), FPG (≤ 6.1, > 6.1 mmol/L), eGFR (< 60, ≥ 60 ml/min/1.73m^2^), HDL-c(< 1, ≥ 1 mmol/L), ALT (≤ 40, > 40U/L), and GLB (< 30, ≥ 30 g/L) [51] were converted to a categorical variable based on the clinical cut point. Secondly, in addition to the stratification factor itself, we adjusted each stratification for all factors (age, sex, BMI, AST, eGFR, ALB, ALT, GLB, GGT, HDL-c, DBIL, BUN, TG, FBG, TC, TB, and LDL-c). Lastly, tests for interaction were performed with the likelihood ratio test of models with and without interaction terms [52, 53].

##### Sensitivity analysis

We performed a series of sensitivity analyses to test our results' robustness [54]. The SUA/Scr ratio was transformed into a categorical variable by quintiles and calculated the P for the trend to validate the SUA/Scr ratio as the continuous variable and test for the possibility of non-linearity. As the risk of NAFLD was obviously increased in patients with diabetes mellitus [10], IFG [55], and elevated ALT [56]. Therefore, when exploring the association between the SUA/Scr ratio and NAFLD in other sensitivity analyses, we excluded participants with FPG > 6.1 mmol/L or ALT > 40 U/L. Since the level of serum uric acid was affected by kidney function, we divided the study population into two groups with impaired kidney function or normal kidney function. As for sensitivity analysis, the relationship between SUA/Scr ratio and NAFLD risk was analyzed in the two populations separately. Besides, we also used a GAM to insert the continuity covariate into the equation (model III) as a curve to ensure the robustness of the results [57]. Additionally, we explored the potential for unmeasured confounding between the SUA/Scr ratio and the risk of NAFLD by calculating E-values [58].

##### Diagnostic value of the SUA/Scr ratio for NAFLD

Using the area under the receiver operating characteristic (ROC) curve (AUC) and its 95% confidence intervals, the overall diagnostic accuracy of the SUA/Scr ratio was determined. We further used the ROC curve to analyze the performance of the SUA/Scr ratio and determined the optimal cut-off value based on the Youden Index. At the same time, the corresponding specificity(SP), sensitivity(SE), negative predictive value (NPV), and positive predictive value (PPV) were calculated.

All results were written according to the STROBE statement [49]. Modeling was performed using the statistical packages R (http://www.R-project.org, The R Foundation) and EmpowerStats (http://www.empowerstats.com, X&Y Solutions, Inc, Boston, MA). *P* values < 0.05 (two-sided) were considered statistically significant.

## Results

### Characteristics of participants

Table 1 provided the demographic and clinical characteristics of participants included in the study. 182,320 adults were included in the final analysis, of whom 90,305 (49.53%) were male and had a mean age of 40.96 ± 14.05 years. The mean SUA/Scr ratio was 3.62 ± 0.92, the mean BMI was 21.42 ± 2.13 kg/m^2^, and the mean LDL-c was 2.25 ± 0.47 mmol/L. The prevalence of NAFLD was 13.66% (24,912/182320). The authors assigned the adults into subgroups using SUA/Scr ratio quintiles (< 2.8, ≥ 2.8 to < 3.3, ≥ 3.3 to < 3.8, ≥ 3.8 to < 4.3, ≥ 4.3). When we set the SUA/Scr ratio < 2.8 as a reference, the higher value of BMI, GGT, ALB, ALT, eGFR, TB, UA, TG, DBIL, AST, TC, BUN, LDL-c were detected in the groups of SUA/Scr ratio ≥ 4.3. Besides, the group (SUA/Scr ratio ≥ 4.3) had a higher proportion of men.

**Fig. 2:**
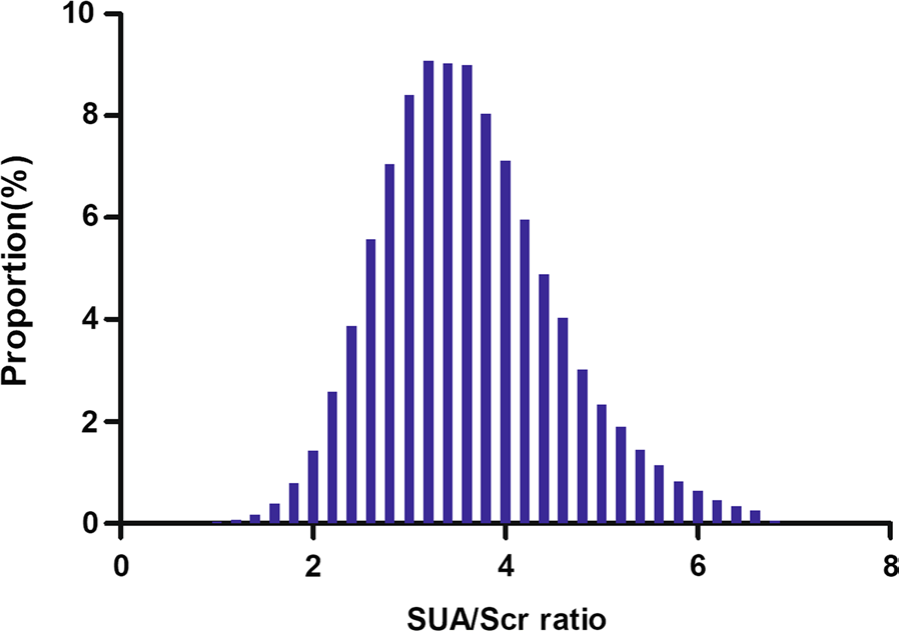
Distribution of SUA/Scr ratio. Figure 2 It presented a normal SUA/Scr ratio distribution while being in the range from 0.574 to 6.738

**Fig. 3:**
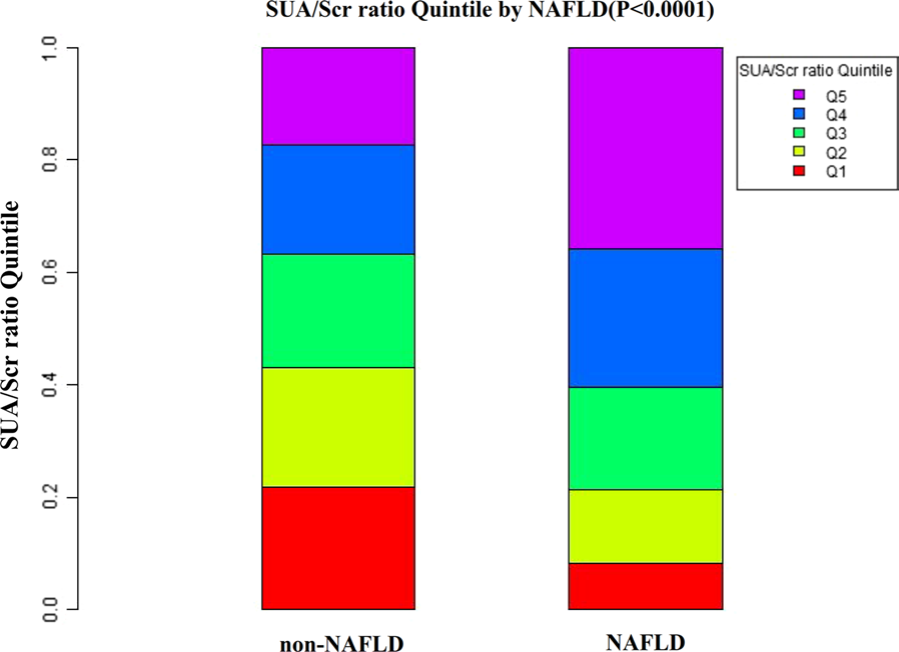
The SUA/Scr ratio levels of all participants from the NAFLD and non-NAFLD groups. Figure 3 indicated that the distribution level of the SUA/Scr ratio in the NAFLD group was higher. In contrast, the SUA/Scr ratio level in the non-NAFLD group was relatively lower

**Fig. 4:**
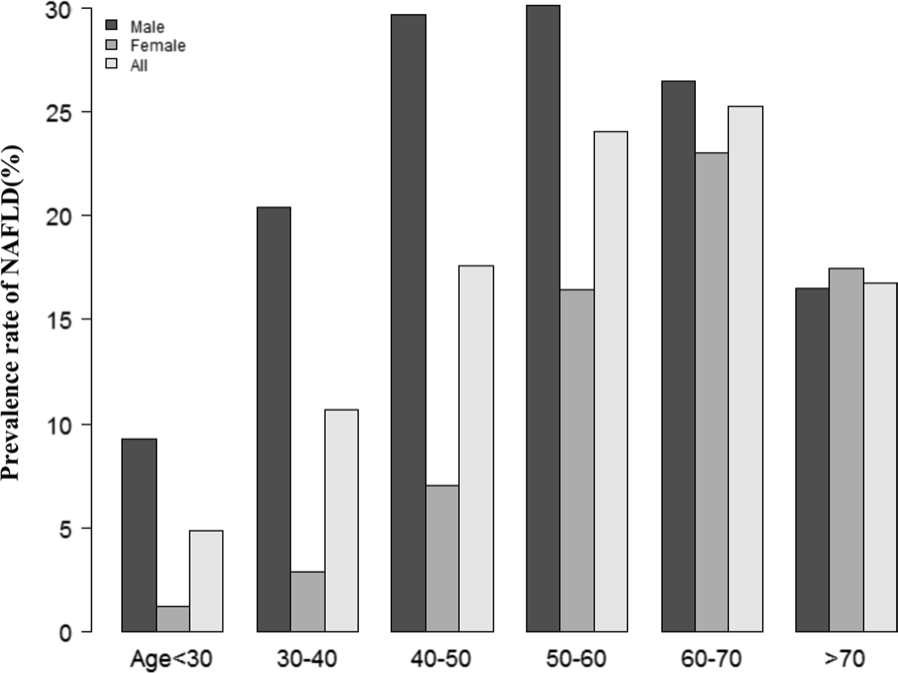
NAFLD prevalence of age stratification by 10 intervals. Figure 4 showed that in age stratification by 10 intervals, except for age > 70, male subjects had a higher prevalence of NAFLD than female subjects no matter what age group they were in. It could also be found that the prevalence of NAFLD increased with age, both in male (except for age > 60) and female (except for age > 70) participants

Figure 2 showed the distribution of SUA/Scr ratio levels. It presented a normal distribution in the range from 0.574 to 6.738, with an average of 3.62. Participants were divided into two groups based on whether they had NAFLD or not. The SUA/Scr ratio values in the two groups were shown in Fig. 3. The results indicated that the distribution level of the SUA/Scr ratio in the NAFLD group was higher. In contrast, the SUA/Scr ratio level in the non-NAFLD group was relatively lower. In age stratification by 10 intervals, except for age > 70, male subjects had a higher prevalence of NAFLD than female subjects no matter what age group they were in (Fig. 4). It also found that the prevalence of NAFLD increased with age, both in male (except for age > 60) and female (except for age > 70) participants.

### The prevalence rate of NALFD

Table 2 revealed that 24,912 participants had NAFLD in total. The total prevalence rate of all participants was 13.66% (13.51%–13.82%). Specifically, the prevalence rates of the five SUA/Scr ratio groups were 5.67% (5.43%–5.91%), 8.86% (8.57%–9.15%), 12.48% (12.14%–12.82%), 16.71% (16.33%–17.09%), and 24.60% (24.16%–25.04%), respectively. Participants with higher SUA/Scr ratios had a higher prevalence of NAFLD compared to the lower SUA/Scr ratio group (*p* < 0.0001 for trend) (Additional file 1: Figure S1).

**Table 2:** Prevalence rate of NAFLD

SUA/Scr ratio	Participants (n)	NAFLD (n)		Prevalence rate (95% CI) (%)
Total	182,320		24,912	13.66(13.51–13.82)
Q1	36,464		2067	5.67(5.43–5.91)
Q2	36,458		3229	8.86(8.57–9.15)
Q3	36,459		4550	12.48(12.14–12.82)
Q4	36,471		6095	16.71(16.33–17.09)
Q5	36,468		8971	24.60(24.16–25.04)
P for trend				< 0.001

### The results of univariate analyses using a binary logistic regression model

The univariate analysis was conducted on the available data, showing that male, age, BMI, GGT, ALT, AST, ALB, GLB, TB, BUN, Scr, SUA, SUA/Scr ratio, FPG, TC, TG, and LDL-c were positively linked to the risk of NAFLD, and DBIL, eGFR, and HDL-c were negatively connected with the risk of NAFLD (Saw Additional file 2: Table S1 for detail).

### The results of multivariate analyses using the binary logistic regression model

The authors constructed three models using the binary logistic regression model to investigate the association between the SUA/Scr ratio and NAFLD. In the un-adjusted model (Crude model), an increase of 1 unit of SUA/Scr ratio was related to an 80% increase in NAFLD risk (OR = 1.805, 95%CI 1.779 to 1.831). The results were statistically significant. In the minimally-adjusted model (Model I), when the authors only adjusted for demographic variables, each additional unit of SUA/Scr ratio increase could lead to elevated the risk of NAFLD by 63% (OR = 1.634, 95%CI 1.608 to 1.661). The findings on the link between the SUA/Scr ratio and NAFLD risk obtained from the model were statistically significant. In the fully adjusted model (Model II), each additional SUA/Scr ratio unit was accompanied by a 42% increase in NAFLD risk (OR = 1.424, 95%CI 1.396 to1.454). The distribution of confidence intervals indicated that the link between the SUA/Scr ratio and the risk of NAFLD obtained by the model was reliable (Table 3).

**Table 3:** Relationship between SUA/Scr ratio and NAFLD in different models

Variable	Crude model (OR,95%CI, P)	Model I (OR,95%CI, P)	Model II (OR,95%CI, P)	Model III (OR,95%CI, P)
SUA/Scr	1.805 (1.779, 1.831) < 0.00001	1.634 (1.608, 1.661) < 0.00001	1.424 (1.396, 1.454) < 0.00001	1.392 (1.363, 1.421) < 0.00001
*SUA/Scr (Quintile)*				
Q1	Ref	Ref	Ref	Ref
Q2	1.617 (1.527, 1.712) < 0.00001	1.387 (1.304, 1.475) < 0.00001	1.424 (1.396, 1.454) < 0.00001	1.219 (1.141, 1.302) < 0.00001
Q3	2.373 (2.248, 2.505) < 0.00001	1.836 (1.732, 1.947) < 0.00001	1.607 (1.510, 1.710) < 0.00001	1.489 (1.397, 1.588) < 0.00001
Q4	3.339 (3.169, 3.518) < 0.00001	2.461 (2.325, 2.605) < 0.00001	1.954 (1.837, 2.078) < 0.00001	1.771 (1.662, 1.886) < 0.00001
Q5	5.429 (5.162, 5.710) < 0.00001	3.800 (3.597, 4.015) < 0.00001	2.609 (2.449, 2.780) < 0.00001	2.378 (2.228, 2.537) < 0.00001
P for trend	< 0.00001	< 0.00001	< 0.00001	< 0.00001

Crude model: we did not adjust other covariants

Model I: we adjusted age, sex, BMI

Model II: we adjusted age, sex, BMI, ALT, AST, GGT, ALB, GLB, DBIL, BUN, FBG, TG, HDL-c, LDL-c, TB, eGFR

Model III: we adjusted age(smooth), sex, BMI (smooth), ALT (smooth), AST (smooth), ALB (smooth), GLB (smooth), DBIL (smooth), BUN (smooth), FBG (smooth), TC (smooth), TG (smooth), HDL-c (smooth), LDL-c(smooth), TB (smooth), eGFR (smooth)

*OR* odds ratios; *CI* confidence, *Ref* reference; *SUA/Scr* serum uric acid-to-creatinine ratio

### Sensitivity analysis

A series of sensitivity analyses were performed to confirm our findings' robustness. The authors converted the SUA/Scr ratio from a continuous variable to a categorical variable (according to quintile) and then put the categorical-transformed SUA/Scr ratio back into the model. The results showed that after the SUA/Scr ratio was transformed into a categorical variable, the trend of the effect sizes in different groups was equidistant, and P for the trend was consistent with the result when the SUA/Scr ratio was a continuous variable (Tables 3 and 4).

**Table 4:** Relationship between UA/Scr ratio and NAFLD in different sensitivity analyses

Exposure	Model I (OR,95%CI, P)	Model II (OR,95%CI, P)	Model III (OR,95%CI, P)	Model IV (OR,95%CI, P)
SUA/Scr	1.445 (1.414, 1.477) < 0.00001	1.413 (1.382, 1.444) < 0.00001	1.428 (1.399, 1.458) < 0.00001	1.278 (1.121, 1.456) 0.00023
*SUA/Scr (Quintile)*				
Q1	Ref	Ref	Ref	Ref
Q2	1.308 (1.217, 1.404) < 0.00001	1.268 (1.184, 1.358) < 0.00001	1.281 (1.198, 1.370) < 0.00001	1.161 (0.908, 1.484) 0.23352
Q3	1.662 (1.552, 1.779) < 0.00001	1.587 (1.485, 1.695) < 0.00001	1.596 (1.497, 1.703) < 0.00001	1.512 (1.159, 1.973) 0.00230
Q4	2.028 (1.895, 2.170) < 0.00001	1.914 (1.793, 2.043) < 0.00001	1.944 (1.824, 2.072) < 0.00001	1.735 (1.277, 2.358) 0.00043
Q5	2.735 (2.552, 2.932) < 0.00001	2.546 (2.380, 2.724) < 0.00001	2.611 (2.446, 2.787) < 0.00001	1.843 (1.211, 2.804) 0.00430
P for trend	< 0.00001	< 0.00001	< 0.00001	0.00001

Model I was sensitivity analysis in participants without FPG > 6.1 mmol/L (n = 171,532). We adjusted age, sex, BMI, eGFR, ALT, AST, GGT, ALB, GLB, DBIL, TB, BUN, FBG, TG, HDL-c, LDL-c

Model II was sensitivity analysis in participants without ALT > 40U/L (n = 169,898). We adjusted age, sex, BMI, eGFR, ALT, AST, GGT, ALB, GLB, DBIL, TB, BUN, FBG, TG, HDL-c, LDL-c

Model III was sensitivity analysis in participants with eGFR ≥ 60 ml/min/1.73m^2^ (n = 178,481). We adjusted age, sex, BMI, eGFR, ALT, AST, GGT, ALB, GLB, DBIL, TB, BUN, FBG, TG, HDL-c, LDL-c

Model IV was sensitivity analysis in participants with eGFR < 60 ml/min/1.73m^2^ (n = 3839). We adjusted age, sex, BMI, eGFR, ALT, AST, GGT, ALB, GLB, DBIL, TB, BUN, FBG, TG, HDL-c, LDL-c

*OR* odds ratios; *CI* confidence, *Ref* reference; *SUA/Scr* serum uric acid-to-creatinine ratio

In addition, the authors used a GAM to insert the continuity covariate into the equation as a curve. The result of Model III showed this generally remained consistent with the fully adjusted model (OR = 1.392, 95%CI: 1.363–1.421, *P* < 0.00001) (Table 3). Besides, the authors generated an E-value to assess the sensitivity to unmeasured confounding. The E-value was 2.20. The E-value was greater than the relative risk of unmeasured confounders and the SUA/Scr ratio, suggesting unmeasured or unknown confounders had little effect on the relationship between the SUA/Scr ratio and NAFLD risk.

Furthermore, the authors excluded participants with FPG > 6.1 mmol/L in other sensitivity analyses. 6289 (3.45%) participants considered prediabetes, and 4649 (2.55%) considered diabetes. The results suggested that after adjusting the confounding factors, the SUA/Scr ratio was also positively associated with NAFLD risk (OR = 1.445, 95% CI:1.414 to 1.477) (Table 4). The authors also excluded participants with ALT > 40U/L for sensitivity analyses. The results suggested that after adjusting age, BMI, sex, ALT, ALB, HDL-c, AST, GLB, eGFR, DBIL, TG, GGT, BUN, FBG, TB, and LDL-c, the SUA/Scr ratio was still positively associated with NAFLD (OR = 1.413, 95% CI:1.382 to 1.444) (Table 4). Since the level of SUA was affected by kidney function, we divided the study population into two groups: impaired or normal kidney function, when we analyzed the relationship between SUA/Scr ratio and NAFLD risk. The results showed that the SUA/Scr ratio was positively associated with NAFLD in impaired and normal kidney function participants (Table 4).

### The nonlinearity addressed by the generalized additive model

**Fig. 5:**
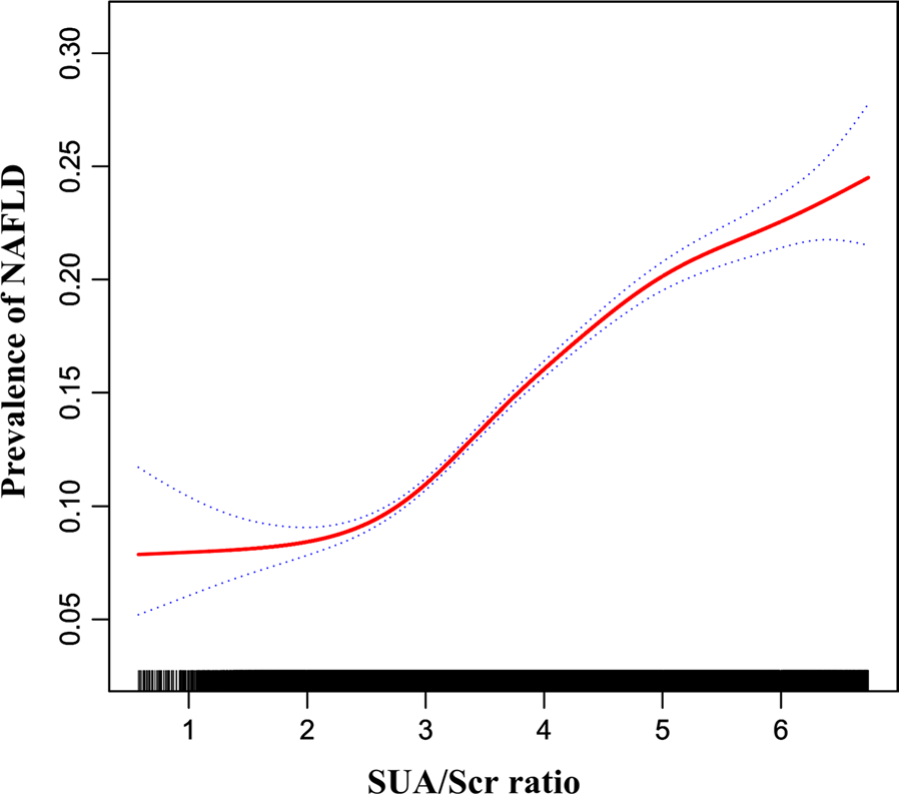
The non-linear relationship between SUA/Scr ratio and the risk of NAFLD. A non-linear relationship was detected after adjusting for age, sex, BMI, ALT, AST, GGT, eGFR, ALB, GLB, DBIL, BUN, FBG, TB, TG, HDL-c, and LDL-c

**Fig. 6:**
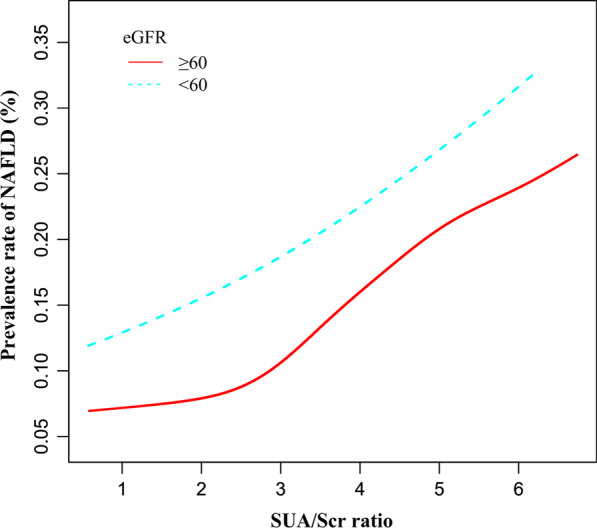
The non-linear relationship between SUA/Scr ratio and the risk of NAFLD in participants with impaired kidney function and normal kidney function. A non-linear relationship was detected in participants with normal kidney function. However, the non-linear relationship was not apparent in participants with impaired kidney function

Through the GAM and smooth curve fitting, we observed that the association between SUA/Scr ratio and NAFLD risk was non-linear (Fig. 5). Therefore, fit the data into a piecewise binary logistic regression model to fit two different slopes. The authors also fit data by standard binary logistic regression model based on the sensitivity analysis and selected the best fit model through the log-likelihood ratio test (Table 5). In the present study, *P* < 0.05 for the log-likelihood ratio test. We, on that account, used a two-piecewise model to fitting the link between SUA/Scr ratio and NAFLD risk. The authors obtained the inflection point as 4.43 through a recursive algorithm and then calculated the effect size and confidence intervals around the inflection point through a two-piece binary logistic regression model. On the left side of the inflection point, the effect size and 95%CI were 1.535, 1.490, and 1.582, respectively. On the right side of the inflection point, the effect size and 95%CI were 1.242, 1.187, and 1.299, respectively. Simultaneously, when analyzing the non-linear relationship between the SUA/Scr ratio and NAFLD risk, we also analyzed two populations with impaired and normal kidney function, respectively. The results suggested there was still a non-linear relationship between the SUA/Scr ratio and NAFLD in participants with normal kidney function. The inflection point of the SUA/Scr ratio was 4.425. On the left side of the inflection point, the OR and 95%CI were 1.511, 1.504, and 1.599, while on the right side, 1.234, 1.179, and 1.291 were measured. However, we further found the *P*-value for the log-likelihood ratio test was not less than 0.05 in the populations with eGFR < 60 ml/min/1.73m^2^. It indicated that there was no non-linear relationship between the SUA/Scr ratio and NAFLD in participants with impaired kidney function (Table 5, Fig. 6).

**Table 5:** The result of the two-piecewise logistic regression model

	Model I (OR,95%CI, P)	Model II (OR,95%CI, P)	Model III (OR,95%CI, P)
Fitting model by standard logistic regression	1.424 (1.396, 1.454) < 0.0001	1.428 (1.399, 1.458) < 0.0001	1.278 (1.121, 1.456) 0.0002
*Fitting model by two-piecewise logistic regression*			
Inflection point of SUA/Scr	4.43	4.425	2.685
≤ Inflection point	1.535 (1.490, 1.582) < 0.0001	1.551 (1.504, 1.599) < 0.0001	0.967 (0.700, 1.336) 0.8403
> Inflection point	1.242 (1.187, 1.299) < 0.0001	1.234 (1.179, 1.291) < 0.0001	1.417 (1.194, 1.681) < 0.0001
P for the log-likelihood ratio test	< 0.001	< 0.001	0.070

Model I: All participants; Model II: Participants with eGFR ≥ 60 ml/min/1.73m^2^; Model III: Participants with eGFR < 60 ml/min/1.73m^2^

*OR* odds ratios; *CI* confidence, *Ref* reference; *UA/Scr* serum uric acid-to-creatinine ratio

We adjusted age, sex, BMI, ALT, eGFR, AST, GGT, ALB, GLB, DBIL, BUN, FBG, TG, TB, HDL-c, LDL-c

### The results of subgroup analyses

The authors performed a subgroup analysis to consider other influencing factors, such as eGFR and FPG, that might affect the results on the relationship between the SUA/Scr ratio and the risk of NAFLD. The authors used gender, age, FPG, BMI, HDL-c, ALT, eGFR, and GLB as the stratification variables to detect the trend of effect sizes in these variables (Table 6). Table 6 showed that gender, age, FPG, BMI, and HDL-c could modify the relationship between the SUA/Scr ratio and NAFLD risk (All P for interaction < 0.05). And a stronger association was observed in females (OR = 1.653, 95%CI: 1.597–1.712) and the population with HDL-c ≥ 1 mmol/L (OR = 1.460, 95%CI: 1.429–1.492), FPG ≤ 6.1 mmol/L (OR = 1.435, 95%CI: 1.404–1.466), BMI < 18.5 kg/m^2^ (OR = 2.032, 95%CI: 1.449–2.848), and BMI between 18.5 and 24 kg/m^2^ (OR = 1.543, 95%CI: 1.508–1.579). A stronger relationship could also be observed among those younger than 30 years (OR = 1.553, 95%CI: 1.469–1.642) and those between the ages of 30 to 40 years (OR = 1.539, 95%CI: 1.484–1.596), and 40 and 50 years (OR = 1.436, 95%CI: 1.387–1.487). In contrast, the weaker association was probed in males (OR = 1.321, 95%CI: 1.290–1.353), and the population with FPG > 6.1 mmol/L (OR = 1.199, 95%CI: 1.135–1.266), HDL-c < 1 mmol/L (OR = 1.247, 95%CI: 1.186–1.311), and BMI ≥ 24 kg/m^2^ (OR = 1.344, 95%CI: 1.297–1.394). The weaker relationship could also be observed among those between the ages of 50 to 60 years (OR = 1.405, 95%CI: 1.350–1.463), 60 to 70 years (OR = 1.341, 95%CI: 1.267–1.420), and above 70 years (OR = 1.381, 95%CI: 1.297–1.471).

**Table 6:** Effect size of SUA/Scr on NAFLD in prespecified and exploratory subgroups

Characteristic	No of participants	OR (95%CI) *P* value P for interaction
Age, years < 30 30 to 40 40 to 50 50 to 60 60 to 70	42,177 56,690 41,167 22,149 10,007	< 0.0001 1.553 (1.469, 1.642) < 0.0001 1.539 (1.484, 1.596) < 0.0001 1.436 (1.387, 1.487) < 0.0001 1.405 (1.350, 1.463) < 0.0001 1.341 (1.267, 1.420) < 0.0001
≥ 70	10,130	1.381 (1.297, 1.471) < 0.0001
Gender		< 0.0001
Male	90,305	1.321 (1.290, 1.353) < 0.0001
Female	92,015	1.653 (1.597, 1.712) < 0.0001
BMI (kg/m^2^)		< 0.0001
< 18.5	18,085	2.032 (1.449, 2.848) < 0.0001
≥ 18.5, < 24	140,638	1.543 (1.508, 1.579) < 0.0001
≥ 24	23,597	1.344 (1.297, 1.394) < 0.0001
FPG (mmol/L)		< 0.0001
≤ 6.1	171,532	1.435 (1.404, 1.466) < 0.0001
> 6.1	10,788	1.199 (1.136, 1.266) < 0.0001
HDL-c (mmol/L)		< 0.0001
< 1	14,221	1.247 (1.186, 1.311) < 0.0001
≥ 1	168,099	1.460 (1.429, 1.492) < 0.0001
ALT (U/L)		0.8957
≤ 40	169,898	1.406 (1.377, 1.436) < 0.0001
> 40	12,422	1.401 (1.332, 1.474) < 0.0001
GLB (g/L)		0.2555
< 30	107,345	1.414 (1.379, 1.449) < 0.0001
≥ 30	74,975	1.443 (1.402, 1.485) < 0.0001
eGFR (ml/min/1.73m^2^)		0.0804
< 60	3839	1.235 (1.099, 1.388) 0.0004
≥ 60	178,481	1.373 (1.348, 1.398) < 0.0001

Note 1: Above model adjusted for age, sex, BMI, eGFR, ALT, AST, GGT, ALB, GLB, DBIL, BUN, FBG, TG, TB, HDL-c, LDL-c

Note 2: In each case, the model is not adjusted for the stratification variable

*OR* odds ratios; *CI* confidence, *Ref* reference; *UA/Scr* serum uric acid-to-creatinine ratio

### Diagnostic value of the SUA/Scr ratio for NAFLD

**Fig. 7:**
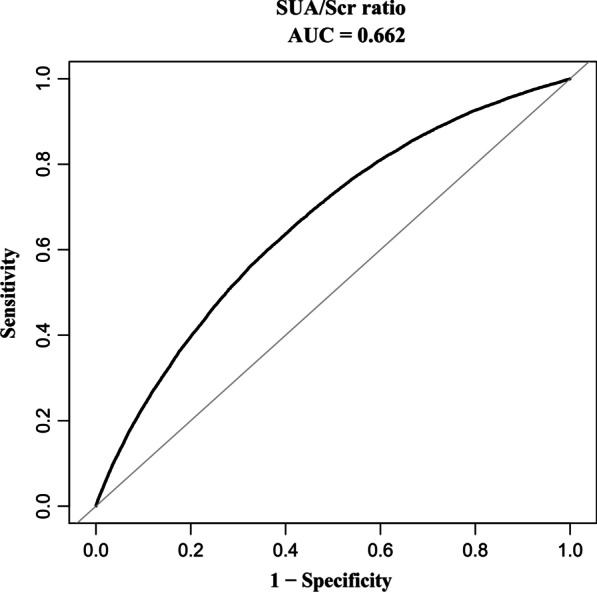
The ROC curve of the SUA/Scr ratio for identifying NAFLD. Figure 6 The diagnostic accuracy of the SUA/Scr ratio in separating participants with and without NAFLD was analyzed by using the ROC method. The SUA/Scr ratio had an AUC of 0.6624 (95% CI 0.6589, 0.6660)

The studies above found that the SUA/Scr ratio and NAFLD were independently associated. We further analyzed the diagnostic value of the SUA/Scr ratio for NAFLD. We applied the ROC method to analyze the diagnostic accuracy of the SUA/Scr ratio for detecting NAFLD. The SUA/Scr ratio had an AUC of 0.6624 (95% CI 0.6589, 0.6660). When the cut-off point of the SUA/Scr ratio was set at 3.776 to discriminate NAFLD, it would meet the highest Youden’s index (0.2377) and the diagnostic accuracy of sensitivity (59.8%)/specificity (64.0%), and PPV (20.8%)/NPV (91.0%). Since the negative predictive value was up to 91%, there was a 91% chance that a patient with the SUA/Scr ratio below 3.776 would not have NAFLD (Table 7, Fig. 7).

**Table 7:** Diagnostic accuracy of the SUA/Scr ratio for identifying NAFLD

	AUC	95%CI low	95%CI upp	Cut-off	SP (%)	SE (%)	PPV (%)	NPV (%)	Youden’s index
the SUA/Scr ratio	0.6624	0.6589	0.6660	3.776	59.8	64.0	20.8	91.0	0.2377

*PPV* Positive predictive value; *SP* specificity; *NPV* Negative predictive value; *SE* Sensitivity; *SUA/Scr ratio* serum uric acid-to-creatinine ratio

## Discussion

The present cross-sectional study was designed to analyze the association between the SUA/Scr ratio and NAFLD in Chinese non-obese people with a normal range of LDL-c. The authors found that the increase of the SUA/Scr ratio was related to a significantly increased risk of NALFD. In addition, a threshold effect curve was also found, and different relationships of the SUA/Scr ratio on NAFLD were detected on both sides of the inflection point in participants with normal kidney function. Besides, age, sex, BMI, FPG, and HDL-c were found as the potential effect modifiers to modify the relationship between SUA/Scr ratio and the risk of NAFLD, as significantly stronger associations were observed in females and the population with age < 50 years, BMI < 24 kg/m^2^, FPG ≤ 6.1 mmol/L, and HDL-c ≥ 1 mmol/L, while significantly weaker association were detected in males and the people with age ≥ 50 years, BMI ≥ 24 kg/m^2^, FPG > 6.1 mmol/L, and HDL-c < 1 mmol/L. In addition, the SUA/Scr ratio has a certain reference value for determining NAFLD.

The estimated prevalence rate of NAFLD ranges from 25 to 31% in the general population of China in recent years [3, 4]. However, the prevalence of NAFLD in the present study was only 13.66%, lower than the reported level. To explore the reason, the participants in this study were non-obese people with a normal range of LDL-c, both of which are risk factors for NAFLD [59, 60]. This made it acceptable that participants in this study had a lower prevalence of NAFLD than the general population. It is worth noting that even in such a population, the prevalence of NAFLD was still 13.66%, especially in men and people older than 40 years old. Therefore, it was still imperative to actively look for other risk factors leading to NAFLD.

Through a literature search, the authors found only two pieces of literature currently investigating the relationship between the SUA/Scr ratio and NAFLD risk [35, 36]. Both studies were cross-sectional studies. One study included 778 subjects who participated in a health examination at the Wonkwang University Hospital Health Promotion Center in South Korea, with a mean SUA/Scr ratio of 6.37 ± 1.65 and a mean LDL-c level of 6.77 ± 1.85. The study found that when the SUA/Scr ratio increased by 1 unit, the risk of NAFLD increased by 18.2% (OR = 1.182, 95% CI: 1.066–1.311) after adjusting for sex, DBP, smoking, SBP, FPG, ALT, TC, AST, HDL-c, GGT, TG, and LDL-c. Another study in China included 282 individuals with normal SUA levels and different glucose tolerance statuses from the Diabetes Program. NAFLD participants' mean BMI, LDL-C, and SUA/Scr ratio were 28.42 ± 3.83 kg/m2, 3.08 ± 0.71 mmol/L, 4.56 ± 1.51, respectively, and the median FPG level was 6.09 mmol/L. The logistic regression analysis was used to explore the association of the SUA/Scr ratio with NAFLD risk. The results suggested that SUA/Scr ratio was positively related to the risk of NAFLD (OR: 2.288, 95% CI: 1.592–3.288) after adjustment for age, BMI, gender, waist circumference (WC), TG, SBP, HDL-c, homeostasis model assessment of insulin resistance, glucose tolerance status, LDL-c, AST and AST. In the present cross-sectional study, having a larger sample size, the logistic regression model showed a positive association between the SUA/Scr ratio and the risk of NAFLD, consistent with those two studies. However, compared with the other two studies, BMI, LDL-C, FPG, and SUA/Scr ratio levels were lower in the present cross-sectional study (Table 1, Table S2). The reason was considered to be related to the limitation of the present cross-sectional study to the non-obese population with a normal range of LDL-c. Studies have reported that the SUA/Scr ratio level was positively correlated with BMI and LDL-c [36], and FPG was positively correlated with BMI and LDL-c [61]. The findings of our study extend the existing literature that the SUA/Scr ratio increases the risk of NAFLD regardless of whether participants had normal or abnormal levels of BMI, LDL-c, FPG, SUA, etc. In addition, the covariates adjusted for the present cross-sectional study differed from those of the other two studies. We adjusted more biochemical parameters, including ALB, GLB, DBIL, and BUN. Evidence showed that those parameters were associated with NAFLD [62, 63]. Meanwhile, the sensitivity analysis found that this relationship still existed among participants without FPG > 6.1 mmol/L or ALT > 40U/L and in persons with either impaired or normal kidney function. The efforts as mentioned above have confirmed the relationship's stability between the SUA/Scr ratio and NALFD risk. The results provided a reference for clinical intervention in SUA/Scr ratio levels to reduce the risk of NALFD.

Furthermore, to the best of our knowledge, the present cross-sectional study observed a non-linear relationship between the SUA/Scr ratio and NAFLD risk for the first time. In the current study, the authors used a two-piecewise logistic regression model to clarify a non-linear relation between the SUA/Scr ratio and the risk of NAFLD in participants with normal kidney function. The inflection point was 4.425 after adjusting for confounders. It showed that when SUA/Scr ratio was below 4.425, a 1 unit increase in the SUA/Scr ratio level was associated with a 55.1% greater adjusted OR of the risk of NAFLD (OR = 1.551, 95%CI: 1.504–1.599). However, when SUA/Scr ratio > 4.425, a 1 unit increase in the SUA/Scr ratio level was only associated with a 23.4% greater adjusted OR of the risk NAFLD (OR = 1.234, 95%CI: 1.179–1.291). The reason may be that other variables other than the SUA/Scr ratio also affected NAFLD risk. It could be seen from Table S3 that compared with the SUA/Scr ratio < 4.425 group, people with SUA/Scr ratio ≥ 4.425 have generally higher levels of BMI, ALT, ALB, BUN, TC, AST, TG, GGT, LDL-c, and a higher proportion of males. However, the above indicators were closely related to NAFLD [51, 60, 64–66]. When the SUA/Scr ratio was greater than 4.425, due to the presence of these NAFLD risk factors, SUA/Scr ratio had a relatively weak effect on NAFLD risk. On the contrary, when SUA/Scr ratio was less than 4.425, the level of the risk factors for NAFLD, such as BMI, GGT, TG, AST, ALB, BUN, TC, ALT, LDL-c was lower, and the impact on NAFLD was weakened, at this time the effect of the SUA/Scr ratio was relatively enhanced. However, our results also found that in participants with impaired kidney function, the non-linear relationship between the SUA/Scr ratio and NAFLD was not significant, which may be related to the reason that the level of uric acid and the prevalence of NAFLD are influenced by renal dysfunction.

Although the underlying mechanism between SUA/Scr ratio and NAFLD is still not fully understood, numerous studies have shown a strong relationship between SUA and NAFLD [67, 68]. SUA might act as a pro-oxidant and interact with oxidants, thereby inducing the oxidative stress responses and production of free radicals [69], which are critical factors in developing NAFLD. Thus, SUA may directly affect NAFLD as a pro-oxidant [70]. Our findings provide an essential rationale for preventing NAFLD by intervening in the SUA/Scr ratio level in the clinic, especially when the SUA/Scr ratio is below 4.425 in participants with normal kidney function. Because when the SUA/Scr ratio is lower than 4.425, the risk of NAFLD might decrease significantly. In patients with normal kidney function, when lowering the SUA/Scr ratio levels below 4.425, continuing to lower the SUA/Scr ratio levels might be more effective in reducing the risk of NAFLD. In addition, in patients with impaired kidney function, although renal function affects the SUA/Scr ratio levels and the risk of NAFLD, controlling serum uric acid levels by drugs and other means is still effective in reducing the risk of NAFLD.

The authors found that age, gender, BMI, FPG, and HDL-c could serve as the potential effect modifiers to modify the relationship between the SUA/Scr ratio and NAFLD in subgroup analysis. Stronger associations were observed in females and those with age < 50 years, FPG ≤ 6.1 mmol/L, BMI < 24 kg/m^2^, and HDL-c ≥ 1 mmol/L. In comparison, significantly weaker associations were detected in males and the population with age ≥ 50 years, BMI ≥ 24 kg/m^2^, FPG > 6.1 mmol/L, and HDL-c < 1 mmol/L. This study and previous studies have confirmed that males, age ≥ 40 years, BMI ≥ 24 kg/m^2^, FPG > 6.1 mmol/L, and HDL-c < 1 mmol/L are closely associated with increased risk of NAFLD [55, 71–73]. Therefore, it is not surprising that the association of SUA/Scr ratio with NAFLD in males and the population with age ≥ 50 years, BMI ≥ 24 kg/m^2^, FPG > 6.1 mmol/L, and HDL-c < 1 mmol/L is weakened by the influence of these factors themselves on NAFLD. Since these factors could modify the relationship between the SUA/Scr ratio and NAFLD, it is clinically possible to reduce the risk of NAFLD by altering the strength of the association between the SUA/Scr ratio and NAFLD through interfering with HDL, FPG, and BMI levels.

The present cross-sectional study has some strengths, and we listed them as follows. An advantage of our research is the large sample size that allows for such analyses, whereas most previous studies were limited to small sample sizes. To our knowledge, this is the first time to observe the association between the SUA/Scr ratio and NAFLD in Chinese non-obese people with a normal range of low-density lipoprotein cholesterol. Compared to prior studies, the study of nonlinear addressing is a considerable advance. Multiple imputation was employed to handle missing data. This method could maximize statistical power and minimize potential bias caused by covariate information missing. The study was observational and is susceptible to potential confounding. But the authors use strict statistical adjustments to minimize residual confounders. The robustness of this study was tested with a set of sensitivity analyses (target independent variable transformation, subgroup analysis, using a GAM to insert the continuity covariate into the equation as a curve, calculating E-values to explore the potential for unmeasured confounding, and reanalyzing the association between the SUA/Scr ratio and NAFLD in participants with normal and impaired kidney function, after excluding participants with FPG > 6.1 mmol/L, or ALT > 40U/L) to ensure the reliability of the results.

The present research has the following shortcomings and needs attention. First, the design of this study is a cross-sectional study, so it cannot get the exact causal relationship because of the nature of the cross-sectional design. Second, the findings can be generalized to Chinese non-obese people with only a normal range of LDL-c. The relationship of SUA/Scr ratio on NAFLD might be different in participants with BMI > 25 or LDL-c > 3.12 mmol/L. In the future, we can consider designing our studies and collecting all the participants, including normal weight and obese patients, with normal and abnormal LDL-c levels. Therefore, we can explore the relationship between the SUA/Scr ratio and NAFLD at different BMI and LDL-c levels. Third, as with all observational studies, although known potential confounders such as blood pressure, FPG, and TG were controlled, there may still be uncontrolled or unmeasured confounders, such as antihypertensive, lipid-lowering, uric acid, or antidiabetic agents. However, the authors calculated the E-value to quantify the potential impact of unmeasured confounders and found that unmeasured confounders were unlikely to explain the results. In addition, when we design our research in the future, we can consider designing our studies and collecting more variables, including antihypertensive, lipid-lowering, SUA-lowering, or antidiabetic agents, waist circumference, hip circumference, serum insulin, etc. Therefore, we can explore the relationship between the SUA/Scr ratio and NAFLD, considering the effect of the medicine on SUA/Scr. We could also compare the diagnostic or predictive value of different models for NAFLD, such as fatty liver index (FLI), ZJU index, and hepatic steatosis index (HIS). Finally, in this study, the diagnosis of NAFLD was made by ultrasonography rather than biopsy. This might have reduced the accuracy of the results. Furthermore, ultrasonography cannot differentiate steatosis from steatohepatitis. However, ultrasonography to diagnose NAFLD has been widely used in epidemiological studies [74].

## Conclusion

This study demonstrates an independent positive association between the SUA/Scr ratio and NAFLD in Chinese non-obese people with a normal range of low-density lipoprotein cholesterol. There is also a non-linear relationship between the SUA/Scr ratio and NAFLD in participants with normal kidney function, and the SUA/Scr ratio is strongly related to NAFLD when SUA/Scr ratio is less than 4.425. This result is expected to provide a reference for the clinicians to control SUA/Scr ratio. It makes sense to reduce the SUA/Scr ratio level could reduce the risk of NAFLD no matter in patients with impaired or normal kidney function. In addition, the SUA/Scr ratio has a certain reference value for determining NAFLD. When the SUA/Scr ratio is lower than 3.776, there is tremendous clinical confidence in identifying a patient as low-risk NAFLD without requiring further clarification by ultrasonography.

## Supplementary Information

**Additional file 1. Figure S1**. Prevalence of NAFLD according to the quintiles of SUA/Scr ratio.

**Additional file 2. Table S1**. The results of univariate analysis.** Table S2**. The characteristics of participants between NAFLD and non-NAFLD groups.** Table S3**. The characteristics of participants on both sides of the inflection point.

## Data Availability

Data can be downloaded from the ‘DATADRYAD’ database (www.Datadryad.org).

## Abbreviations

NAFLD: Non-alcoholic fatty liver disease
FPG: Fasting plasma glucose
NASH: Non-alcoholic steatohepatitis
BMI: Body mass index
Scr: Serum creatinine
UA: Uric acid
SUA: Serum uric acid
SUA/Scr ratio: Serum uric acid-to-creatinine ratio
TC: Total cholesterol
BUN: Serum urea nitrogen
TG: Triglyceride
ALT: Alanine aminotransferase
ALB: Albumin
HDL-c: High-density lipoprotein cholesterol
AST: Aspartate aminotransferase
LDL-c: Low-density lipoprotein cholesterol
DBIL: Direct bilirubin
GLB: Globulin
GGT: γ-Glutamyl transpeptidase
TB: Total bilirubin
WC: Waist circumference
OR: Odds ratio
Ref: Reference
CI: Confidence intervals
T2DM: Type 2 diabetes mellitus
COPD: Chronic obstructive pulmonary disease
CVD: Cardiovascular disease
GAM: Generalized additive model
CKD: Chronic kidney disease
WHO: World Health Organization
MAR: Missing-at-random
AUROC: Area under the receiver-operating characteristic curve
ROC: Receiver operating characteristic
AUC: Area under the ROC curve
SP: Specificity
SE: Sensitivity
NPV: Negative predictive value
PPV: Positive predictive value
IFG: Impaired fasting glucose
SD: Standard deviation

## References

[1] Schuppan D, Schattenberg JM (2013). Non-alcoholic steatohepatitis: pathogenesis and novel therapeutic approaches. J Gastroenterol Hepatol.

[2] Zhang H, Chen L, Xin Y, Lou Y, Liu Y, Xuan S (2014). Apolipoprotein c3 gene polymorphisms are not a risk factor for developing non-alcoholic Fatty liver disease: a meta-analysis. Hepatmon.

[3] Wong VW, Chan WK, Chitturi S, Chawla Y, Dan YY, Duseja A, Fan J, Goh KL, Hamaguchi M, Hashimoto E (2018). Asia-pacific working party on non-alcoholic fatty liver disease guidelines 2017-part 1: definition, risk factors and assessment. J Gastroenterol Hepatol.

[4] National Workshop on Fatty Liver and Alcoholic Liver Disease, Chinese Society of Hepatology, Chinese Medical Association; Fatty Liver Expert Committee, Chinese Medical Doctor Association. [Guidelines of prevention and treatment for nonalcoholic fatty liver disease: a 2018 update]. Zhonghua Gan Zang Bing Za Zhi. 2018;26(3):195–203. Chinese. 10.3760/cma.j.issn.1007-3418.2018.03.008. 10.3760/cma.j.issn.1007-3418.2018.03.008 PMC12769340 29804393

[5] Targher G, Byrne CD, Lonardo A, Zoppini G, Barbui C (2016). Non-alcoholic fatty liver disease and risk of incident cardiovascular disease: a meta-analysis. J Hepatol.

[6] Mantovani A, Zaza G, Byrne CD, Lonardo A, Zoppini G, Bonora E, Targher G (2018). Nonalcoholic fatty liver disease increases risk of incident chronic kidney disease: a systematic review and meta-analysis. Metabolism.

[7] Mantovani A, Byrne CD, Bonora E, Targher G (2018). Nonalcoholic fatty liver disease and risk of incident type 2 diabetes: a meta-analysis. Diabetes Care.

[8] Zheng X, Cao C, He Y, Wang X, Wu J, Hu H (2021). Association between nonalcoholic fatty liver disease and incident diabetes mellitus among Japanese: a retrospective cohort study using propensity score matching. Lipids Health Dis.

[9] Zhang M, Lin S, Wang MF, Huang JF, Liu SY, Wu SM, Zhang HY, Wu ZM, Liu WY, Zhang DC (2020). Association between NAFLD and risk of prevalent chronic kidney disease: why there is a difference between east and west?. Bmc Gastroenterol.

[10] Rinella ME (2015). Nonalcoholic fatty liver disease: a systematic review. JAMA.

[11] Jiang T, Chen X, Xia C, Liu H, Yan H, Wang G, Wu Z (2019). Association between Helicobacter pylori infection and non-alcoholic fatty liver disease in North Chinese: a cross-sectional study. Sci Rep.

[12] Mikolasevic I, Milic S, Turk WT, Grgic I, Jakopcic I, Stimac D, Wensveen F, Orlic L (2016). Nonalcoholic fatty liver disease - A multisystem disease?. World J Gastroenterol.

[13] Yang MH, Sung J, Gwak GY (2016). The associations between apolipoprotein B, A1, and the B/A1 ratio and nonalcoholic fatty liver disease in both normal-weight and overweight Korean population. J Clin Lipidol.

[14] Sookoian S, Pirola CJ (2017). Systematic review with meta-analysis: risk factors for non-alcoholic fatty liver disease suggest a shared altered metabolic and cardiovascular profile between lean and obese patients. Aliment Pharmacol Ther.

[15] Sporea I, Popescu A, Dumitrașcu D, Brisc C, Nedelcu L, Trifan A, Gheorghe L, Fierbințeanu BC (2018). Nonalcoholic fatty liver disease: status quo. J Gastrointest Liver Dis.

[16] Polyzos SA, Kountouras J, Mantzoros CS (2019). Obesity and nonalcoholic fatty liver disease: from pathophysiology to therapeutics. Metabolism.

[17] Younossi ZM, Stepanova M, Negro F, Hallaji S, Younossi Y, Lam B, Srishord M (2012). Nonalcoholic fatty liver disease in lean individuals in the United States. Medicine.

[18] Fan JG, Kim SU, Wong VW (2017). New trends on obesity and NAFLD in Asia. J Hepatol.

[19] VanWagner LB, Armstrong MJ (2018). Lean NAFLD: a not so benign condition?. Hepatol Commun.

[20] Hagström H, Nasr P, Ekstedt M, Hammar U, Stål P, Hultcrantz R, Kechagias S (2018). Risk for development of severe liver disease in lean patients with nonalcoholic fatty liver disease: a long-term follow-up study. Hepatol Commun.

[21] Fukuda T, Hamaguchi M, Kojima T, Hashimoto Y, Ohbora A, Kato T, Nakamura N, Fukui M (2016). The impact of non-alcoholic fatty liver disease on incident type 2 diabetes mellitus in non-overweight individuals. LIVER INT.

[22] Kim SS, Cho HJ, Kim HJ, Kang DR, Berry JR, Kim JH, Yang MJ, Lim SG, Kim S, Cheong JY (2018). Nonalcoholic fatty liver disease as a sentinel marker for the development of diabetes mellitus in non-obese subjects. Dig Liver Dis.

[23] Yoshitaka H, Hamaguchi M, Kojima T, Fukuda T, Ohbora A, Fukui M (2017). Nonoverweight nonalcoholic fatty liver disease and incident cardiovascular disease: a post hoc analysis of a cohort study. Medicine.

[24] Gaggini M, Morelli M, Buzzigoli E, DeFronzo RA, Bugianesi E, Gastaldelli A (2013). Non-alcoholic fatty liver disease (NAFLD) and its connection with insulin resistance, dyslipidemia, atherosclerosis and coronary heart disease. Nutrients.

[25] Imajo K, Hyogo H, Yoneda M, Honda Y, Kessoku T, Tomeno W, Ogawa Y, Taguri M, Mawatari H, Nozaki Y (2014). LDL-migration index (LDL-MI), an indicator of small dense low-density lipoprotein (sdLDL), is higher in non-alcoholic steatohepatitis than in non-alcoholic fatty liver: a multicenter cross-sectional study. PLoS ONE.

[26] Sun DQ, Wu SJ, Liu WY, Wang LR, Chen YR, Zhang DC, Braddock M, Shi KQ, Song D, Zheng MH (2016). Association of low-density lipoprotein cholesterol within the normal range and NAFLD in the non-obese Chinese population: a cross-sectional and longitudinal study. BMJ Open.

[27] Sun DQ, Liu WY, Wu SJ, Zhu GQ, Braddock M, Zhang DC, Shi KQ, Song D, Zheng MH (2016). Increased levels of low-density lipoprotein cholesterol within the normal range as a risk factor for nonalcoholic fatty liver disease. Oncotarget.

[28] Li JJ, Huang YH, Lin YY, Li MM, Chen YF, Cai RW (2017). The association of uric acid with leukoaraiosis. J Int Med Res.

[29] Xu X, Li C, Zhou P, Jiang T (2016). Uric acid transporters hiding in the intestine. Pharm Biol.

[30] Bardin T, Richette P (2017). Impact of comorbidities on gout and hyperuricaemia: an update on prevalence and treatment options. BMC Med.

[31] Darmawan G, Hamijoyo L, Hasan I (2017). Association between serum uric acid and non-alcoholic fatty liver disease: a meta-analysis. Acta Med Indones.

[32] Al-Daghri NM, Al-Attas OS, Wani K, Sabico S, Alokail MS (2017). Serum uric acid to creatinine ratio and risk of metabolic syndrome in Saudi type 2 diabetic patients. Sci Rep.

[33] Gu L, Huang L, Wu H, Lou Q, Bian R (2017). Serum uric acid to creatinine ratio: a predictor of incident chronic kidney disease in type 2 diabetes mellitus patients with preserved kidney function. Diab Vasc Dis Res.

[34] Durmus KN, Sasak G, Aka AU, Akgun M, Boga S, Sengul A, Gungor S, Arinc S (2016). Serum uric acid levels and uric acid/creatinine ratios in stable chronic obstructive pulmonary disease (COPD) patients: are these parameters efficient predictors of patients at risk for exacerbation and/or severity of disease?. Med Sci Monit.

[35] Seo YB, Han AL (2021). Association of the serum uric acid-to-creatinine ratio with nonalcoholic fatty liver disease diagnosed by computed tomography. Metab Syndr Relat Disord.

[36] Ma C, Liu Y, He S, Zeng J, Li P, Ma C, Ping F, Zhang H, Xu L, Li W (2020). C-Peptide: a mediator of the association between serum uric acid to creatinine ratio and non-alcoholic fatty liver disease in a chinese population with normal serum uric acid levels. Front Endocrinol.

[37] Zhang N, Hu X, Zhang Q, Bai P, Cai M, Zeng TS, Zhang JY, Tian SH, Min J, Huang HT (2018). Non-high-density lipoprotein cholesterol: high-density lipoprotein cholesterol ratio is an independent risk factor for diabetes mellitus: Results from a population-based cohort study. J Diabetes.

[38] Steventon JJ, Trueman RC, Ma D, Yhnell E, Bayram-Weston Z, Modat M, Cardoso J, Ourselin S, Lythgoe M, Stewart A (2016). Longitudinal in vivo MRI in a Huntington's disease mouse model: Global atrophy in the absence of white matter microstructural damage. Sci Rep.

[39] Zeng MD, Li YM, Chen CW, Lu LG, Fan JG, Wang BY, Mao YM (2008). Guidelines for the diagnosis and treatment of alcoholic liver disease. J Dig Dis.

[40] Wang J, Xie P, Huang JM, Qu Y, Zhang F, Wei LG, Fu P, Huang XJ (2016). The new Asian modified CKD-EPI equation leads to more accurate GFR estimation in Chinese patients with CKD. Int Urol Nephrol.

[41] Alberti KG, Zimmet PZ (1998). Definition, diagnosis and classification of diabetes mellitus and its complications. Part 1: diagnosis and classification of diabetes mellitus provisional report of a WHO consultation. Diabet Med.

[42] Shamu T, Chimbetete C, Egger M, Mudzviti T (2021). Treatment outcomes in HIV infected patients older than 50 years attending an HIV clinic in Harare, Zimbabwe: a cohort study. PLoS ONE.

[43] Ding Q, Xu L, Zhu Y, Xu B, Chen X, Duan Y, Xie Z, Shen K (2020). Comparison of clinical features of acute lower respiratory tract infections in infants with RSV/HRV infection, and incidences of subsequent wheezing or asthma in childhood. BMC Infect Dis.

[44] Rifkin DE, Katz R, Chonchol M, Fried LF, Cao J, de Boer IH, Siscovick DS, Shlipak MG, Sarnak MJ (2010). Albuminuria, impaired kidney function and cardiovascular outcomes or mortality in the elderly. Nephrol Dial Transplant.

[45] Wu SJ, Zou H, Zhu GQ, Wang LR, Zhang Q, Shi KQ, Han JB, Huang WJ, Braddock M, Chen YP (2015). Increased levels of systolic blood pressure within the normal range are associated with significantly elevated risks of nonalcoholic fatty liver disease. Medicine.

[46] Wu SJ, Zhu GQ, Ye BZ, Kong FQ, Zheng ZX, Zou H, Shi KQ, Lin L, Braddock M, Huang WJ (2015). Association between sex-specific serum uric acid and non-alcoholic fatty liver disease in Chinese adults: a large population-based study. Medicine.

[47] Groenwold RH, White IR, Donders AR, Carpenter JR, Altman DG, Moons KG (2012). Missing covariate data in clinical research: when and when not to use the missing-indicator method for analysis. CMAJ.

[48] White IR, Royston P, Wood AM (2011). Multiple imputation using chained equations: Issues and guidance for practice. Stat Med.

[49] von Elm E, Altman DG, Egger M, Pocock SJ, Gøtzsche PC, Vandenbroucke JP (2014). The strengthening the reporting of observational studies in epidemiology (STROBE) statement: guidelines for reporting observational studies. Int J Surg.

[50] Rothenbacher D, Rehm M, Iacoviello L, Costanzo S, Tunstall-Pedoe H, Belch J, Söderberg S, Hultdin J, Salomaa V, Jousilahti P (2020). Contribution of cystatin C- and creatinine-based definitions of chronic kidney disease to cardiovascular risk assessment in 20 population-based and 3 disease cohorts: the BiomarCaRE project. BMC Med.

[51] Wu L, Zhang M, Hu H, Wan Q (2021). Elevated gamma-glutamyl transferase has a non-linear association with incident non-alcoholic fatty liver disease in the non-obese Chinese population: a secondary retrospective study. Lipids Health Dis.

[52] Mullee A, Romaguera D, Pearson-Stuttard J, Viallon V, Stepien M, Freisling H, Fagherazzi G, Mancini FR, Boutron-Ruault MC, Kühn T (2019). Association between soft drink consumption and mortality in 10 European Countries. JAMA Intern Med.

[53] Keidel D, Anto JM, Basagaña X, Bono R, Burte E, Carsin AE, Forsberg B, Fuertes E, Galobardes B, Heinrich J et al. the role of socioeconomic status in the association of lung function and air pollution-a pooled analysis of three adult escape cohorts. Int J Environ Res Public Health 2019; 16(11). 10.3390/ijerph16111901 PMC6603717 31146441

[54] Sun D, Li W, Zhang H, Li Y, Zhang Q (2020). Inverted U-shaped relationship between body mass index and multivessel lesions in Chinese patients with myocardial infarction: a cross-sectional study. J Int Med Res.

[55] Vesa CM, Behl T, Nemeth S, Bratu OG, Diaconu CC, Moleriu RD, Negrut N, Zaha DC, Bustea C, Radu FI (2020). Prediction of NAFLD occurrence in prediabetes patients. Exp Ther Med.

[56] Shengir M, Krishnamurthy S, Ghali P, Deschenes M, Wong P, Chen T, Sebastiani G (2020). Prevalence and predictors of nonalcoholic fatty liver disease in South Asian women with polycystic ovary syndrome. World J Gastroenterol.

[57] Zhu F, Chen C, Zhang Y, Chen S, Huang X, Li J, Wang Y, Liu X, Deng G, Gao J (2020). Elevated blood mercury level has a non-linear association with infertility in U.S. women: data from the NHANES 2013–2016. Reprod Toxicol.

[58] Haneuse S, VanderWeele TJ, Arterburn D (2019). Using the E-value to assess the potential effect of unmeasured confounding in observational studies. JAMA.

[59] Zhou M, Yang N, Xing X, Chang D, Li J, Deng J, Chen Y, Hu C, Zhang R, Lu X (2021). Obesity interacts with hyperuricemia on the severity of non-alcoholic fatty liver disease. BMC Gastroenterol.

[60] Xu C, Yu C, Ma H, Xu L, Miao M, Li Y (2013). Prevalence and risk factors for the development of nonalcoholic fatty liver disease in a nonobese Chinese population: the Zhejiang Zhenhai Study. AM J Gastroenterol.

[61] Wang J, Zhang Y, Liu Z, Yang Y, Zhong Y, Ning X, Zhang Y, Zhao T, Xia L, Geng F (2020). Schizophrenia patients with a metabolically abnormal obese phenotype have milder negative symptoms. BMC Psychiatry.

[62] Ge P, Yang H, Lu J, Liao W, Du S, Xu Y, Xu H, Zhao H, Lu X, Sang X (2016). Albumin binding function: the potential earliest indicator for liver function damage. Gastroenterol Res Pract.

[63] Tian J, Zhong R, Liu C, Tang Y, Gong J, Chang J, Lou J, Ke J, Li J, Zhang Y (2016). Association between bilirubin and risk of non-alcoholic fatty liver disease based on a prospective cohort study. Sci Rep.

[64] Akhavan RA, Dadgar MM, Ghasemi NM, Shirazinia M, Ghodsi H, Rouhbakhsh ZM, Tavakolizadeh NM, Hoseini B, Akhavan RK (2018). Association between smoking and non-alcoholic fatty liver disease: a systematic review and meta-analysis. SAGE Open Med.

[65] Noureddin M, Loomba R (2012). Nonalcoholic fatty liver disease: Indications for liver biopsy and noninvasive biomarkers. Clin Liver Dis.

[66] Amor AJ, Perea V (2019). Dyslipidemia in nonalcoholic fatty liver disease. Curr Opin Endocrinol Diabetes Obes.

[67] Shih MH, Lazo M, Liu SH, Bonekamp S, Hernaez R, Clark JM (2015). Association between serum uric acid and nonalcoholic fatty liver disease in the US population. J Formos Med Assoc.

[68] Yuan H, Yu C, Li X, Sun L, Zhu X, Zhao C, Zhang Z, Yang Z (2015). Serum uric acid levels and risk of metabolic syndrome: a dose-response meta-analysis of prospective studies. J Clin Endocrinol Metab.

[69] Lin KM, Lu CL, Hung KC, Wu PC, Pan CF, Wu CJ, Syu RS, Chen JS, Hsiao PJ, Lu KC (2019). The paradoxical role of uric acid in osteoporosis. Nutrients.

[70] Spahis S, Delvin E, Borys JM, Levy E (2017). Oxidative stress as a critical factor in nonalcoholic fatty liver disease pathogenesis. Antioxid Redox Signal.

[71] Jian C, Xu Y, Ma X, Shen Y, Wang Y, Bao Y (2020). Neck circumference is an effective supplement for nonalcoholic fatty liver disease screening in a community-based population. Int J Endocrinol.

[72] Alsabaani AA, Mahfouz AA, Awadalla NJ, Musa MJ, Al HS (2018). Non-alcoholic fatty liver disease among type-2 diabetes mellitus patients in Abha City, South Western Saudi Arabia. Int J Environ Res Public Health.

[73] Hu X, Huang Y, Bao Z, Wang Y, Shi D, Liu F, Gao Z, Yu X (2012). Prevalence and factors associated with nonalcoholic fatty liver disease in Shanghai work-units. BMC Gastroenterol.

[74] Leite NC, Villela-Nogueira CA, Cardoso CR, Salles GF (2014). Non-alcoholic fatty liver disease and diabetes: from physiopathological interplay to diagnosis and treatment. World J Gastroenterol.

