# Supplementary figures and images for: Association between serum uric acid-to-creatinine ratio and non-alcoholic fatty liver disease: a cross-sectional study in Chinese non-obese people with a normal range of low-density lipoprotein cholesterol
Source: BMC Gastroenterol. 2022 Sep 14;22:419. doi: 10.1186/s12876-022-02500-w (PMC9472393; doi:10.1186/s12876-022-02500-w)

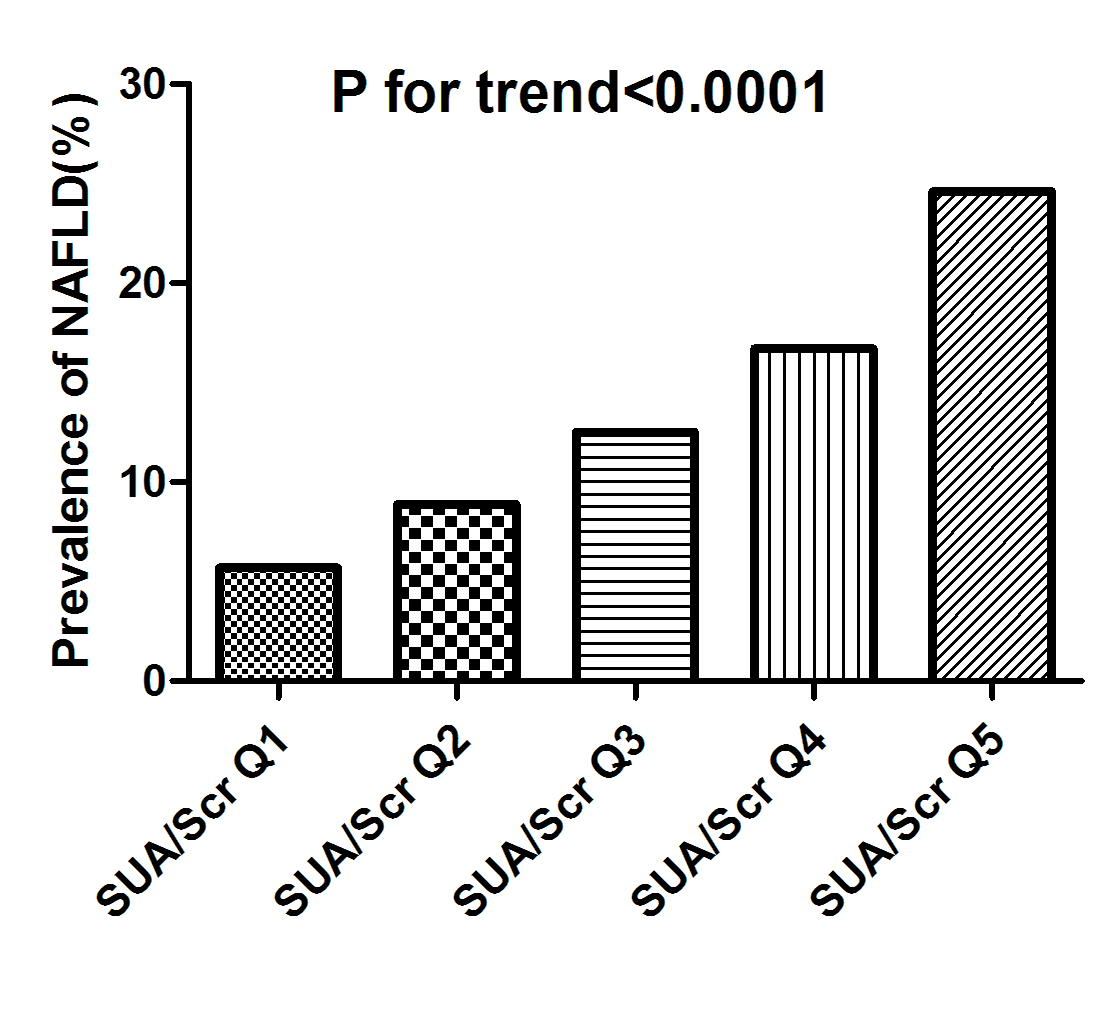

Supplement: Supplementary file 1 — Additional file 1. Figure S1. Prevalence of NAFLD according to the quintiles of SUA/Scr ratio. [file 12876_2022_2500_MOESM1_ESM.tif]
