# Supplementary material for: Association between serum uric acid-to-creatinine ratio and non-alcoholic fatty liver disease: a cross-sectional study in Chinese non-obese people with a normal range of low-density lipoprotein cholesterol
Source: BMC Gastroenterol. 2022 Sep 14;22:419. doi: 10.1186/s12876-022-02500-w (PMC9472393; doi:10.1186/s12876-022-02500-w)
Supplement: Supplementary file 2 — Additional file 2. Table S1. The results of univariate analysis. Table S2. The characteristics of participants between NAFLD and non-NAFLD groups. Table S3. The characteristics of participants on both sides of the inflection point. [file 12876_2022_2500_MOESM2_ESM.docx]

**Association between serum uric acid-to-creatinine ratio and non-alcoholic fatty liver disease: a cross-sectional study in** **Chinese nonobese people with a normal range of low-density lipoprotein cholesterol**

**Running title: The relationship between SUA/Scr ratio and NAFLD**

**Xiaoyu Wang^#1^, Yong Han^#2,3,8^, Yufei Liu^#4,5,8^, Haofei Hu****^*6,7,8^**

^1^Department of Nephrology, Hechi People's Hospital, Hechi 547000, Guangxi Zhuang Autonomous Region, China

^2^Department of Emergency, Shenzhen Second People’s Hospital, Shenzhen 518000, Guangdong Province, China

^3^Department of Emergency, The First Affiliated Hospital of Shenzhen University, Shenzhen 518000, Guangdong Province, China

^4^Department of Neurosurgery, Shenzhen Second People’s Hospital, Shenzhen 518000, Guangdong Province, China

^5^Department of Neurosurgery, The First Affiliated Hospital of Shenzhen University, Shenzhen 518000, Guangdong Province, China

^6^Department of Nephrology, Shenzhen Second People’s Hospital, Shenzhen 518000, Guangdong Province, China

^7^Department of Nephrology, The First Affiliated Hospital of Shenzhen University, Shenzhen 518000, Guangdong Province, China

^8^Shenzhen University Health Science Center, Shenzhen 518000, Guangdong Province, China

**^#^**Xiaoyu Wang, Yong Han, and Yufei Liu have contributed equally to this work.

*Corresponding author

**Haofei HU**

Department of Nephrology,

Shenzhen Second People’s Hospital,

No.3002 Sungang Road, Futian District,

Shenzhen 518000,

Guangdong Province,

China

Tel:+86-755-83366388

E-mail: [huhaofei0319@126.com](mailto:huhaofei0319@126.com)

**Table S1. The results of univariate analysis**

| Variable | Statistics | OR (95%CI) P value |
| --- | --- | --- |
|  |  |  |
| Gender |  |  |
| Female | 92015 (50.469%) | Ref. |
| Male | 90305 (49.531%) | 4.256 (4.125, 4.392) <0.00001 |
| Age, years | 40.964 ± 14.054 | 1.031 (1.030, 1.032) <0.00001 |
| BMI, kg/m^2^ | 21.424 ± 2.125 | 2.087 (2.065, 2.109) <0.00001 |
| GGT, U/L | 27.302 ± 30.652 | 1.020 (1.019, 1.020) <0.00001 |
| ALT, U/L | 19.752 ± 18.131 | 1.033 (1.032, 1.034) <0.00001 |
| AST, U/L | 22.447 ± 11.315 | 1.030 (1.029, 1.031) <0.00001 |
| ALB, g/L | 44.578 ± 2.711 | 1.062 (1.057, 1.067) <0.00001 |
| GLB, g/L | 29.339 ± 3.667 | 1.010 (1.006, 1.014) <0.00001 |
| TB, umol/L | 12.293 ± 5.048 | 1.009 (1.007, 1.012) <0.00001 |
| DBIL, umol/L | 2.005 ± 1.043 | 0.985 (0.972, 0.998) 0.02132 |
| BUN, mmol/L | 4.399 ± 1.265 | 1.178 (1.166, 1.190) <0.00001 |
| Scr, umol/L | 78.640 ± 19.355 | 1.019 (1.019, 1.020) <0.00001 |
| eGFR, ml/min/1.73m^2^ | 100.522 ± 18.324 | 0.981 (0.980, 0.982) <0.00001 |
| UA, umol/L | 281.659 ± 87.106 | 1.009 (1.009, 1.009) <0.00001 |
| SUA/Scr ratio | 3.620 ± 0.922 | 1.805 (1.779, 1.831) <0.00001 |
| FPG, mmol/L | 5.152 ± 0.848 | 1.687 (1.662, 1.713) <0.00001 |
| TC, mmol/L | 4.535 ± 0.738 | 1.725 (1.694, 1.757) <0.00001 |
| TG, mmol/L | 1.322 ± 0.943 | 2.810 (2.765, 2.855) <0.00001 |
| HDL-c, mmol/L | 1.450 ± 0.355 | 0.104 (0.099, 0.109) <0.00001 |
| LDL-c, mmol/L | 2.250 ± 0.473 | 2.536 (2.459, 2.615) <0.00001 |

Values are n (%) or mean±SD

ALB, albumin; ALT, alanine aminotransferase; AST, aspartate aminotransferase; BMI, body mass index; BUN, blood urea nitrogen; Scr, serum creatinine; DBIL, direct bilirubin; TB, total bilirubin; FPG, fasting plasma glucose; GGT, γ-glutamyl transpeptidase; GLB, globulin; HDL-c, high-density lipoprotein cholesterol; LDL-c, low-density lipoprotein cholesterol; TC, total cholesterol; TG, triglyceride; UA, uric acid; UA/Scr ratio, serum uric acid-to-creatinine ratio; eGFR estimated glomerular filtration rate

**Table S2. The characteristics of participants between NAFLD and non-NAFLD groups.**

|  | non-NAFLD | NAFLD | P-value |
| --- | --- | --- | --- |
| N | 157408 | 24912 |  |
| Age (years) | 40.0 ± 14.0 | 46.8 ± 13.3 | <0.001 |
| Gender |  |  | <0.001 |
| Female | 86469 (54.9%) | 5546 (22.3%) |  |
| Male | 70939 (45.1%) | 19366 (77.7%) |  |
| BMI (kg/m^2^) | 21.1 ± 2.1 | 23.4 ± 1.2 | <0.001 |
| GGT (U/L) | 19.0 (14.0-28.0) | 34.0 (24.0-52.0) | <0.001 |
| ALT (U/L) | 15.0 (11.0-22.0) | 24.0 (18.0-35.0) | <0.001 |
| AST (U/L) | 20.0 (17.0-24.9) | 24.0 (20.0-29.0) | <0.001 |
| ALB (g/L) | 44.5 ± 2.7 | 45.0 ± 2.7 | <0.001 |
| GLB (g/L) | 29.3 ± 3.6 | 29.5 ± 3.8 | <0.001 |
| TB (umol/L) | 12.3 ± 5.1 | 12.5 ± 5.0 | <0.001 |
| DBIL (umol/L) | 1.9 (1.3-2.5) | 1.9 (1.3-2.5) | 0.0004 |
| BUN (mmol/L) | 4.4 ± 1.3 | 4.6 ± 1.2 | <0.001 |
| Scr (umol/L) | 77.6 ± 19.2 | 85.3 ± 18.8 | <0.001 |
| eGFR (ml/min/1.73m^2^) | 101.4 ± 18.3 | 94.7 ± 17.2 | <0.001 |
| SUA (umol/L) | 272.2 ± 83.6 | 341.6 ± 84.8 | <0.001 |
| SUA/Scr ratio | 3.5 ± 0.9 | 4.1 ± 0.9 | <0.001 |
| FPG (mmol/L) | 5.1 ± 0.7 | 5.6 ± 1.3 | <0.001 |
| TC (mmol/L) | 4.5 ± 0.7 | 4.8 ± 0.8 | <0.001 |
| TG (mmol/L) | 1.0 (0.8-1.4) | 1.9 (1.3-2.6) | <0.001 |
| HDL-c (mmol/L) | 1.5 ± 0.4 | 1.3 ± 0.3 | <0.001 |
| LDL-c (mmol/L) | 2.2 ± 0.5 | 2.4 ± 0.5 | <0.001 |

Values are n (%) or mean ± SD or median (quartile)

ALB, albumin; ALT, alanine aminotransferase; AST, aspartate aminotransferase; BMI, body mass index; BUN, blood urea nitrogen; Scr, serum creatinine; DBIL, direct bilirubin; TB, total bilirubin; FPG, fasting plasma glucose; GGT, γ-glutamyl transpeptidase; GLB, globulin; HDL-c, high-density lipoprotein cholesterol; LDL-c, low-density lipoprotein cholesterol; TC, total cholesterol; TG, triglyceride; UA, uric acid; SUA/Scr ratio, serum uric acid-to-creatinine ratio; eGFR, estimated glomerular filtration rate.

**Table S3. The characteristics of participants on both sides of the inflection point.**

| SUA/Scr ratio | <4.425 | >=4.425 | P-value |
| --- | --- | --- | --- |
| N | 149258 | 33062 |  |
| Age(years) | 41.0 ± 14.1 | 40.6 ± 13.9 | <0.001 |
| Gender |  |  | <0.001 |
| Female | 78751 (52.8%) | 13264 (40.1%) |  |
| Male | 70507 (47.2% | 19798 (59.9%) |  |
| BMI (kg/m^2^) | 21.3 ± 2.1 | 21.9 ± 2.1 | <0.001 |
| GGT (U/L) | 20.0 (15.0-29.0) | 26.0 (17.0-43.0) | <0.001 |
| ALT (U/L) | 16.0 (11.1-23.0) | 19.0 (13.0-28.4) | <0.001 |
| AST (U/L) | 21.0 (17.2-25.0) | 22.0 (18.0-27.0) | <0.001 |
| ALB (g/L) | 44.4 ± 2.7 | 45.2 ± 2.6 | <0.001 |
| GLB (g/L) | 29.4 ± 3.7 | 29.3 ± 3.7 | <0.001 |
| TB (umol/L) | 12.3 ± 5.0 | 12.4 ± 5.2 | <0.001 |
| DBIL (umol/L) | 1.8 (1.3-2.5) | 2.0 (1.3-2.7) | <0.001 |
| BUN (mmol/L) | 4.4 ± 1.3 | 4.5 ± 1.2 | <0.001 |
| Scr (umol/L) | 79.9 ± 19.8 | 73.0 ± 16.2 | <0.001 |
| eGFR (ml/min/1.73m^2^) | 98.6 ± 18.1 | 109.0 ± 16.9 | <0.001 |
| UA (umol/L) | 262.2 ± 75.1 | 368.7 ± 84.4 | <0.001 |
| FPG (mmol/L) | 5.2 ± 0.8 | 5.1 ± 0.9 | 0.211 |
| TC (mmol/L) | 4.5 ± 0.7 | 4.6 ± 0.8 | <0.001 |
| TG (mmol/L) | 1.0 (0.8-1.4) | 1.3 (0.9-2.1) | <0.001 |
| HDL (mmol/L) | 1.5 ± 0.4 | 1.3 ± 0.4 | <0.001 |
| LDL (mmol/L) | 2.2 ± 0.5 | 2.3 ± 0.5 | <0.001 |

Values are n (%) or mean ± SD or median (quartile)

ALB, albumin; ALT, alanine aminotransferase; AST, aspartate aminotransferase; BMI, body mass index; BUN, blood urea nitrogen; Scr, serum creatinine; DBIL, direct bilirubin; TB, total bilirubin; FPG, fasting plasma glucose; GGT, γ-glutamyl transpeptidase; GLB, globulin; HDL-c, high-density lipoprotein cholesterol; LDL-c, low-density lipoprotein cholesterol; TC, total cholesterol; TG, triglyceride; UA, uric acid; SUA/Scr ratio, serum uric acid-to-creatinine ratio; eGFR, estimated glomerular filtration rate.

**Figure S1. Prevalence of NAFLD according to the quintiles of** **SUA/Scr ratio.**

Fig. S1. Compared with the lowest SUA/Scr ratio group, participants with a high SUA/Scr ratio had a higher prevalence rate of NAFLD (P<0.001 for trend ).
